# Hyperlipidemic hypersensitivity to lethal microbial inflammation and its reversal by selective targeting of nuclear transport shuttles

**DOI:** 10.1038/s41598-021-91395-w

**Published:** 2021-06-07

**Authors:** Yan Liu, Jozef Zienkiewicz, Kelli L. Boyd, Taylor E. Smith, Zhi-Qi Xu, Jacek Hawiger

**Affiliations:** 1 Division of Allergy, Pulmonary and Critical Care Medicine, Department of Medicine, Vanderbilt University School of Medicine, Nashville, TN USA; 2 Department of Veterans Affairs, Tennessee Valley Health Care System, Nashville, TN USA; 3 Department of Pathology, Microbiology and Immunology, Vanderbilt University School of Medicine, Nashville, TN USA; 4 Department of Molecular Physiology and Biophysics, Vanderbilt University School of Medicine, Nashville, TN USA; 5 Vanderbilt University Medical Center, 21st Avenue South, T-1218, MCN, Nashville, TN 37232 USA

**Keywords:** Diseases, Cytokines, Peptides, Chemokines, Infection, Acute inflammation, Chronic inflammation, Animal disease models

## Abstract

Hyperlipidemia, the hallmark of Metabolic Syndrome that afflicts millions of people worldwide, exacerbates life-threatening infections. We present a new evidence for the mechanism of hyperlipidemic hypersensitivity to microbial inflammation caused by pathogen-derived inducer, LPS. We demonstrate that hyperlipidemic animals succumbed to a non-lethal dose of LPS whereas normolipidemic controls survived. Strikingly, survival of hyperlipidemic animals was restored when the nuclear import of stress-responsive transcription factors (SRTFs), Sterol Regulatory Element-Binding Proteins (SREBPs), and Carbohydrate-Responsive Element-Binding Proteins (ChREBPs) was impeded by targeting the nuclear transport checkpoint with cell-penetrating, biselective nuclear transport modifier (NTM) peptide. Furthermore, the burst of proinflammatory cytokines and chemokines, microvascular endothelial injury in the liver, lungs, heart, and kidneys, and trafficking of inflammatory cells were also suppressed. To dissect the role of nuclear transport signaling pathways we designed and developed importin-selective NTM peptides. Selective targeting of the importin α5, ferrying SRTFs and ChREBPs, protected 70–100% hyperlipidemic animals. Targeting importin β1, that transports SREBPs, was only effective after 3-week treatment that lowered blood triglycerides, cholesterol, glucose, and averted fatty liver. Thus, the mechanism of hyperlipidemic hypersensitivity to lethal microbial inflammation depends on metabolic and proinflammatory transcription factors mobilization, which can be counteracted by targeting the nuclear transport checkpoint.

## Introduction

The present-day epidemic of metabolic disorders is linked to hyperlipidemia, hyperglycemia, and other signs of Metabolic Syndrome (MetS). The High-Fat, High-Cholesterol Diet (also known as the Western Diet) contains an abundant amount of fat, cholesterol, protein, carbohydrates, and salt (a typical menu offered in fast food restaurants). Together with a sedentary lifestyle, the HFD contributes to MetS, the prevalent example of obesity, fatty liver, insulin resistance, and atherosclerosis^1^. According to the World Health Organization (WHO), more individuals succumb to cardiovascular diseases globally from MetS than from any other cause^2^. Mounting evidence indicates that the MetS also contributes to microbial, allergic, and autoimmune diseases^3^.

Infections in patients with MetS-linked underlying diseases of the cardiovascular, respiratory, pancreatic (diabetes), hepatobiliary, and renal systems, include the recent outbreaks of COVID-19. They evolve into a more severe and difficult to control stage of microbial inflammation^4–9^. Hence, these patients are more vulnerable to the fatal outcomes of acute respiratory distress syndrome (ARDS), septic cardiomyopathy, microvascular thrombosis (known as Disseminated Intravascular Coagulation), and Acute Kidney Injury. These complications result from the microvascular endothelial injury due to genomic storm that underlies septic shock, the end stage of microbial inflammation^10^. Microbial inflammation is caused by a wide range of infectious agents encompassing bacteria (e.g. Methicillin-Resistant *Staphylococcus* *aureus,* and multidrug-resistant Gram- bacteria), viruses (e.g. influenza, SARS CoV-2), fungi (e.g. *Candida* sp.), and protozoa (e.g. *Malaria* *falciparum*)^11^.

How do MetS-associated diseases predispose the body to more severe and ultimately fatal infections? Hyperlipidemia comprises an elevated level of cholesteryl esters, triglycerides and other lipids in the blood that constitute a risk factor in cardiovascular and hepatobiliary morbidity and mortality worldwide^2,12^. These disorders are mediated by metabolic inflammation, the body’s response to overfeeding and excessive accumulation of metabolites observed in atherosclerosis, gout, homocystinuria, and other diseases^11^. The intracellular accumulation of cholesterol crystals in atherosclerosis and urate crystals in gout is associated with the activation of inflammasomes^13–15^. Hyperlipidemia in LDL receptor-deficient (*ldlr*^*−/−*^) or Lipoprotein E-deficient mice also activates other innate immunity signaling pathways that depend on their mainstays, namely Toll-like Receptors, TLR-2, TLR-3, and TLR-4, along with their intracellular adaptors, MyD88, TRAM, and TRIF^16–18^. Furthermore, hyperlipidemia increases Endoplasmic Reticulum (ER) stress causing Caspase 2-induced processing of metabolic transcription factors (MTFs) SREBPs^19^ (see Fig. S1).

We reasoned that hyperlipidemia, through its activation of Toll-like receptors 2-, 3-, and 4-linked signaling cascades, enhancement of inflammasomes activity, and evoking ER stress response, would lower the threshold for microbial inflammation thereby increasing its intensity and ultimately fatal outcome. We tested this hypothesis in an experimental model of human familial hypercholesterolemia, the *ldlr*^*−/−*^ mice^20^. These mice, fed a High-Fat Diet (HFD) containing abundant fat, cholesterol, protein, carbohydrates, and salt, gained weight and developed hypercholesterolemia, hypertriglyceridemia, hyperglycemia, and atherosclerosis in the coronary sinus as well as the fatty liver, eventually evolving into steatohepatitis^21^. We postulated that hyperlipidemia in *ldlr*^*−/−*^ mice fed HFD, would represent a common albeit abnormal metabolic status predisposing them to the lethal microbial inflammation caused by pathogen-derived inducer, LPS, at a dosage that is non-lethal for normolipidemic animals. We selected LPS because this virulence factor of Gram-negative bacteria is one of the most potent inducers of microbial inflammation^11,22^. Multidrug-resistant Gram-negative bacteria cause septic shock in 62% patients^23^.

Signals evoked by LPS via the interaction of its toxic moiety, Lipid A, with the cognate receptor, TLR-4, are relayed to the cell’s nucleus through the signaling cascades of proinflammatory Stress-Responsive Transcription factors (SRTFs) (Fig. S1)^10,11^. SRTFs encompass NF-κB, AP-1, NFAT, and STAT-1. As depicted in Fig. S1, signaling cascades for individual transcription factors entail different adaptors and signaling intermediates that form signalosomes, such as CARMA 1 in immune cells and CARMA3 in non-immune cells^11^. These signalosomes activate inhibitor IκB kinase (IKK) complex that promotes phosphorylation, ubiquitination, and proteasomal degradation of Inhibitor of NF-κB (IκB). Its removal allows the unmasking of Nuclear Localization Sequence (NLS) on NF-κB family members. Exposed NLS is recognized by nuclear transport adaptor proteins, importins α, that form a complex with importin β1. These nuclear transport shuttles are needed for the crossing of larger transcription factors (> 40kD), such as SRTFs, through the nuclear pores. Thus, the nuclear transport checkpoint is a pivotal step in signaling to the genome by NF-κB, AP-1, NFAT, and STAT-1 in response to proinflammatory cues. We posited that the recognition step at the nuclear transport checkpoint (see Fig. S1) can be dismantled by fragment-designed, cell-penetrating peptides that compete for the NLS binding sites on importin α5^11^. These peptides, termed nuclear transport modifiers (NTMs), stop the proinflammatory transcription factors in their tracks at the nuclear transport checkpoint. Therefore, the inflammatory response is suppressed due to silencing of the inflammatory regulome in the nucleus.

Excessive level of lipids and glucose in blood is caused by overfeeding or inborn metabolic errors. The metabolic products evoke importin β1-mediated signaling to the nucleus of metabolic transcription factors (MTFs) encompassing SREBP1, SREBP2, and ChREBPs among others^11^. Some of them were implicated in potentially lethal end stage of microbial inflammation, such as sepsis. For example, the metabolic profile of sepsis non-survivors is marked by metabolites linked to fatty acid transport and β oxidation, along with gluconeogenesis^24^. These pathways depend on transcriptional regulation, not only by the peroxisome proliferator-activated receptors α, β, and γ, but also by other MTFs such as SREBPs and ChREBPs. Fortuitously, we found that the control of the nuclear transport checkpoint by NTM peptide not only suppressed the expression of proinflammatory genes regulated by SRTFs, but also metabolic genes controlled by SREBPs (recognized by Importin β1) as well as ChREBPs^21^. The latter can be ferried to the nucleus either by importin α5 or importin β1^11^ (see Fig. S1). Therefore, prototypical biselective NTM by controlling the nuclear transport checkpoint suppresses the expression of proinflammatory genes responsible not only for microbial inflammation (regulated by SRTFs) but also at least 9 genes in charge of metabolic inflammation (regulated by MTFs). Hence, overlapping microbial inflammation and metabolic inflammation can be simultaneously controlled using this approach. We embarked on dissecting nuclear transport pathways ferrying SRTFs and MTFs to the genome in hyperlipidemia complicated by lethal microbial inflammation.

## Results

### Hyperlipidemia increases susceptibility to lethal microbial inflammation caused by the pathogen-derived virulence factor, LPS

**Figure 1:**
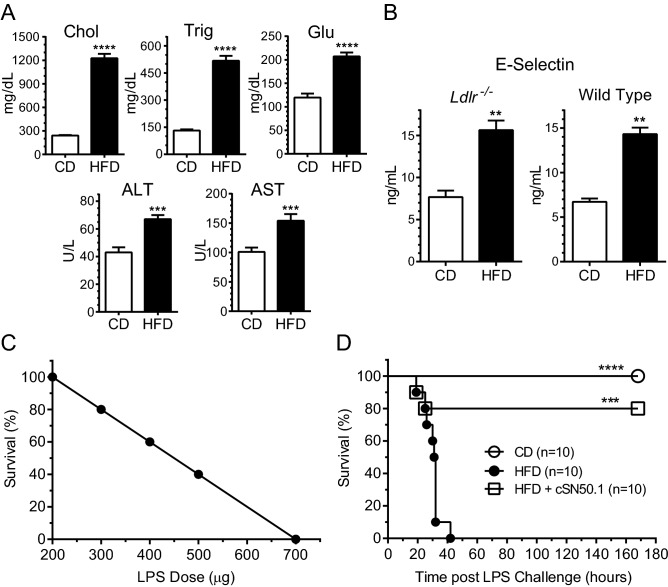
HFD-induced hyperlipidemia and hyperglycemia contribute to microvascular endothelial injury and hypersensitivity to LPS. (**A**,**B**) Elevated blood cholesterol (Chol), triglycerides (Trig), and glucose (Glu) as well as liver transaminases (ALT, AST) in 15-week-old *ldlr*^*−/−*^ mice fed high-fat diet (HFD) for 3 weeks (tested group) or *ldlr*^*−/−*^ mice fed chow diet (CD, control group). In panel (**B**), the effect of HFD on blood marker of microvascular endothelial injury, soluble E-Selectin, in 15-week-old *ldlr*^*−/−*^ mice as compared to age-matched wild type, C57BL/6 mice. Both groups were fed HFD or CD for 3 weeks. (**A**,**B**) Data is presented as a mean ± SEM (n = 10). Statistical significance was determined by nonparametric *t* test with Mann–Whitney rank comparison, **p < 0.005, ***p < 0.0005, ****p < 0.0001. (**C**) LPS-induced death of normolipidemic *ldlr*^*−/−*^ mice is dose-dependent. 15-week-old, chow diet-fed *ldlr*^*−/−*^ female mice (approx. 20 g of weight; n = 10) received a single dose of LPS (200, 300, 400, 500, or 700 μg in 0.2 mL saline) administered IP and 1-week survival was recorded. The survival curve represents a linear correlation with dose of LPS (S = − 0.2 × D_LPS_ + 140; r^2^ = 1.00). (**D**) All hyperlipidemic *ldlr*^*−/−*^ mice succumb to the non-lethal dose of LPS (200 μg) while chow diet-fed cohort survives. Short-term treatment with biselective NTM, cSN50.1 peptide, that targets nuclear import pathways of SRTFs and MTFs, resulted in improved survival (80%). 15-week-old *ldlr*^*−/−*^ female mice (n = 10/group) fed chow diet (CD), fed HFD, and fed HFD and treated with biselective NTM, cSN50.1 peptide (IP, 33 μg/g, 15 doses) given 30 min before LPS challenge (200 μg in 0.2 mL saline) and for three days thereafter. Survival was observed for 7 days. Data is presented as Kaplan–Meier survival plot with *p* value calculated by log rank analysis, ***p < 0.0005, ****p < 0.0001.

Here, we show that the HFD-fed *ldlr*^*−/−*^ mice developed hypercholesterolemia, hypertriglyceridemia, hyperglycemia, and elevated liver transaminases after relatively short (3 weeks) HFD feeding as compared to chow diet-fed mice (Fig. 1A).

Notably, the hyperlipidemic mice displayed an elevated level of the marker of microvascular endothelial injury, soluble Endothelial Selectin (E-Selectin)^25^ in plasma (Fig. 1B). To rule out the potential impact of LDL receptor deficiency on this marker induction, we fed HFD to wild type animals, C57BL/6 mice, with very similar results (Fig. 1B). Thus, hyperlipidemia per se induces the sign of microvascular endothelial injury in wild type and *ldlr*^*−/−*^ mice.

We titrated the LPS response in chow diet-fed, normolipidemic *ldlr*^*−/−*^ mice to determine the highest non-lethal dose of this potent inducer of microbial inflammation. With an increased dose of LPS from 200 to 700 µg (10–35 µg/g) injected intraperitoneally, the 7-day survival declined from 100 to 0%, respectively (Fig. 1C). Therefore, we have used the non-lethal 200 µg (or 10 µg/g) dose of LPS in subsequent experiments.

Strikingly, this dose, which was non-lethal in chow diet-fed *ldlr*^*−/−*^ mice, became deadly in HFD-fed *ldlr*^*−/−*^ mice causing rapid death within 40 h while chow-diet fed *ldlr*^*−/−*^ mice survived for at least 7 days (Fig. 1D). Remarkably, similar to the chow diet-fed *ldlr*^*−/−*^ animals, the 80% survival was observed in HFD-fed *ldlr*^*−/−*^ mice when treated with the bi-selective cell-penetrating NTM, cSN50.1 peptide 30 min before LPS challenge and thereafter for 60 h (see “Short-Term protocol” below) (Fig. 1D). This biselective peptide (Fig. S2A) controls the nuclear transport checkpoint by simultaneously targeting two nuclear transport shuttles, importin α5 (Imp α5) and importin β1 (Imp β1)^21,26^. Importin α5 ferries to the cell’s nucleus the proinflammatory SRTFs, whereas importin β1 is responsible for nuclear translocation of the MTFs, SREBPs^11,27^. To dissect the contribution of each importin-mediated nuclear transport pathway to lethal microbial inflammation in hyperlipidemia, we designed and developed pathway-selective NTM peptides that would target separately importin α5 and importin β1.

### Design, development, and testing of importin pathways-selective nuclear transport modifier peptides

As depicted in Supplementary Fig. 2A, the biselective NTM peptide denoted cSN50.1 is a two-fragment construct comprising the membrane translocating motif based on the Signal Sequence Hydrophobic Region (SSHR), and the Nuclear Localization Sequence (NLS) that is cyclized by the insertion of two cysteines forming the intrachain disulfide bond. The two fragments are derived from the highly conserved human Fibroblast Growth Factor 4 SSHR and human transcription factor NF-κB1 NLS motif, respectively^28^. The importin α5-selective, cell-penetrating peptide termed cSN50.1α (Fig. S2B), has an intact NLS fragment responsible for the interaction with Imp α5 NLS binding pockets^26^. The SSHR fragment has amino acid substitutions, thereby incapacitating its hydrophobic interaction with Imp β1 responsible for translocation of SREBPs to the nucleus^21^. In contrast, the monoselective NTM, cSN50.1β peptide, designed to target Imp β1, has amino acid substitutions within the NLS sequence disabling its docking to Imp α5^26^ (Fig. S2C). The control peptide, Null cSN50.1, contains amino acid substitutions in both fragments, within the SSHR and the NLS motifs, which disabled Null cSN50.1 from targeting Imp β1 and Imp α5 (Fig. S2D). Importantly, the biselective peptide as well as both monoselective and control peptides penetrate the cell membrane. This non-invasive peptide delivery process consists of direct (receptor- and energy-independent) crossing of membrane phospholipid bilayer and bypassing endosomal compartment^29^.

**Figure 2:**
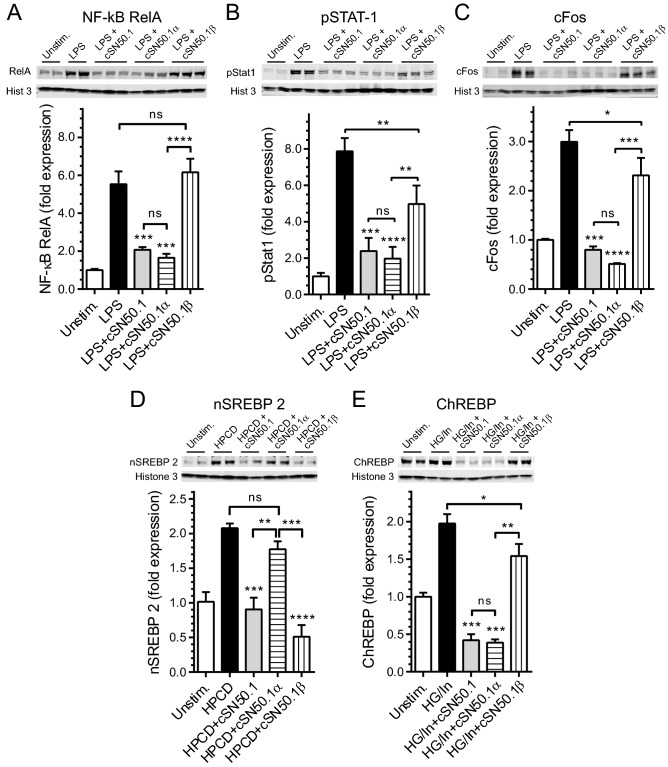
Differential effect of pathway-selective NTM peptides in cultured cells. (**A–C**) Treatment with Imp α5-selective NTM, cSN50.1α inhibits nuclear import of SRTFs: NF-κB RelA (**A**), phosphorylated STAT1 (**B**), and component of AP-1 complex, cFos (**C**) in LPS-stimulated RAW cells. Cells were pretreated with 30 µM NTM peptides (biselective cSN50.1, and two monoselective cSN50.1α and cSN50.1β) for 30 min then incubated with LPS (10 ng/mL) for 6 h. (**D**) Nuclear import of nSREBP2 is inhibited by treatment with Imp β1-selective NTM, cSN50.1β peptide, in sterol-depleted HEK 293T cells. Cells were rapidly depleted of sterols by 15 min incubation with hydroxypropyl-beta-cyclodextrin (HPCD) and treated with NTM peptides (as indicated) for 2 h. (**E**) Metabolic transcription factor (MTF) ChREBP is transported to the nucleus primarily by Imp α5-mediated nuclear import pathway. HepG2 cells were incubated in low glucose (5.5 mM) medium for 24 h. Subsequently, they were treated with high glucose (HG, 25 mM) supplemented with 100 nM insulin (In) to facilitate intracellular influx of glucose. 30 µM NTMs (as indicated) were added and cells were incubated for additional 24 h. (**A**) through (**E**) nuclear content of SRTFs and MTFs was determined by quantitative immunoblotting analysis using LI-COR Odyssey infrared imaging system. Data is presented as a mean ± SEM (n = 4 for **D**,**E**; n = 6 for **A**,**B**,**C**). Statistical significance between LPS-stimulated cells (control) and NTM peptides-treated LPS-stimulated cells was determined by ordinary One-way ANOVA with Holm–Sidak test for multiple comparison, *p < 0.05, **p < 0.005, ***p < 0.0005, ****p < 0.0001. Unedited full-length immunoblots are presented in Supplementary Figure S4.

First, we compared the effect of treatment with two monoselective NTM peptides, cSN50.1α and cSN50.1β, to the effect of treatment with the biselective NTM, cSN50.1 peptide on nuclear translocation of SRTFs and MTFs in cell-based essays. Targeting Imp α5-mediated nuclear import by the monoselective NTM, cSN50.1α peptide, inhibited the nuclear translocation of NF-κB RelA, consistent with previous result in different models of microbial inflammation^30^ (Fig. 2A).

Moreover, we extended this analysis of Imp α5-selective NTM, cSN50.1α peptide inhibition of the nuclear translocation of NF-κB RelA to two other members of the SRTFs set, the phosphorylated form of STAT1, and cFos (Fig. 2B,C). The latter transcription factor is a component of the AP-1 complex comprising cFos and cJun (Fig. S1). In turn, the nuclear translocation of nSREBP2 was spared by selective targeting of Imp α5-mediated nuclear import by cSN50.1α peptide (Fig. 2D). However, somehow surprisingly we found that the monoselective NTM peptide targeting importin α5 also strongly inhibited the nuclear import of ChREBPs in comparison to the lesser effect of cSN50.1β peptide (Fig. 2E). ChREBPs also display bipartite NLS located near phosphorylation site, Ser-196^31^. Thus, we present the new evidence that importin α5 also plays a significant role in the nuclear translocation of ChREBPs, a key regulator of glucose, glycogen, and triglycerides metabolism^32^.

In contrast to Imp α5-selective NTM, cSN50.1α peptide, the biselective cSN50.1 peptide inhibited not only the nuclear import of three tested proinflammatory SRTFs (NFκB RelA, pSTAT1, and cFos), but also two MTFs (nSREBP2 and ChREBP). Furthermore, we showed that monoselective NTM, cSN50.1β peptide, inhibited nuclear transport of nSREBP-2 (Fig. 2D). This agrees with previous studies that identified importin β1 as a sole nuclear import adapter of this MTF^27^. In contrast, inhibition of ChREBPs was not as effective when cells were treated with Imp β1-selective NTM, cSN50.1β peptide (Fig. 2E).

Cumulatively, the selective targeting of the nuclear transport checkpoint in cultured cells indicates that Imp α5 ferries SRTFs and ChREBPs to the nucleus while Imp β1 is solely responsible for the nuclear translocation of SREBPs. The Imp β1-mediated pathway is also involved, albeit to a lesser degree, in ChREBPs nuclear translocation (see Fig. 2E).

We also designed and tested the control “loss of function” NTM, Null cSN50.1 peptide, with disabled binding sites for Imp α5 and Imp β1 (see Fig.S2D). The Null cSN50.1 peptide was tested along with active NTMs in in vivo model of lethal LPS-induced microbial inflammation. Normolipidemic, chow diet-fed, C57BL/6 female mice were challenged with a single lethal dose of LPS (700 μg) administered through IP injection. Mice were treated with active biselective and monoselective NTMs (cSN50.1, cSN50.1α, and cSN50.1β) and two controls (vehicle/saline and Null cSN50.1 peptide) also administered through IP injection. As documented in Fig.S3, mice challenged with the lethal dose of LPS and treated with saline (vehicle) or the control NTM, Null cSN50.1 peptide, died within 24 h. Likewise, Imp β1-selective NTM, cSN50.1β peptide, was not protective in this lethal microbial inflammation-mediated model of endotoxemia (Fig. S3A). In a striking contrast, the 80% survival was observed in these normolipidemic mice when treated with the bi-selective NTM, cSN50.1 peptide or Imp α5-selective NTM, cSN50.1α peptide. Consistent with survival data, blood levels of cytokines TNF-α and IL-6, and chemokine MCP-1 were suppressed in survivors treated with cSN50.1 and cSN50.1α peptides. Contrariwise, these proinflammatory biomarkers remained elevated in mice treated with the control NTM, Null cSN50.1 peptide, and comparable with the levels observed in mice treated with saline (vehicle). As expected, plasma levels of TNF-α, IL-6, and MCP-1 in mice treated with Imp β1-selective NTM, cSN50.1β peptide, were similar to those treated with vehicle and control Null NTM peptide.

### Importin pathway-selective NTM peptides prevent and/or reverse lethal microbial inflammation in hyperlipidemic *ldlr*^*−/−*^ mice

**Figure 3:**
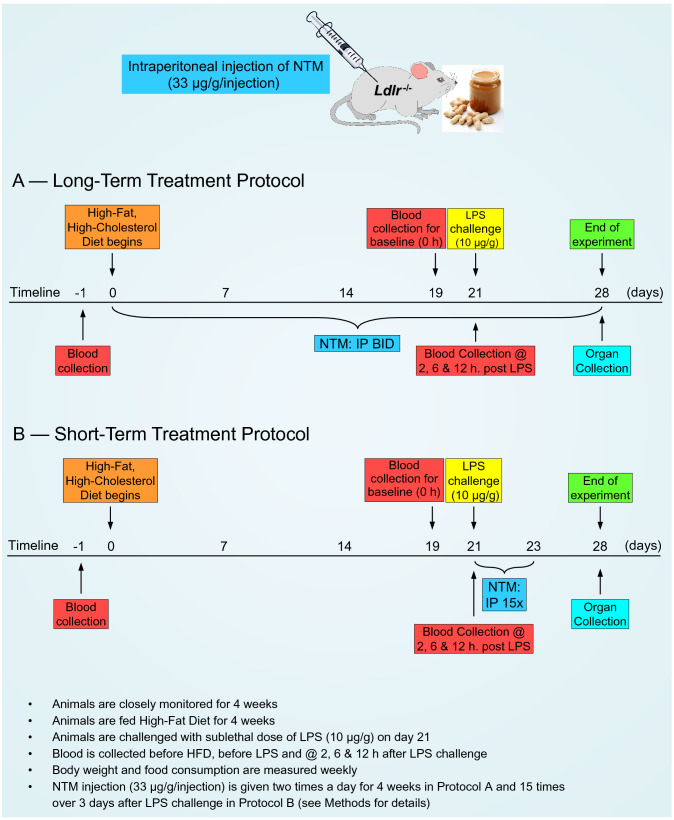
Graphic depiction of two treatment protocols. (**A**) Long-term treatment protocol. 12-week-old *ldlr*^*−/−*^ female mice were placed on HFD and treated with saline (IP, twice a day (BID), 0.2 mL) or NTM peptides (IP, BID, 33 μg/g in 0.2 mL saline) for three weeks before non-lethal dose of LPS (10 μg/g in 0.2 mL saline) was administered through IP injection. HFD feeding and treatment with saline or NTM were continued for additional week. (**B**) Short-term treatment protocol. 15-week-old *ldlr*^*−/−*^ female mice fed HFD for three weeks were challenged with a non-lethal dose of LPS (10 μg/g in 0.2 mL saline, IP) and threated with 15 doses of saline (0.2 mL) or NTM peptides (IP, 33 μg/g in 0.2 mL saline) given 30 min before LPS challenge (10 μg/g in 0.2 mL saline) and for three days thereafter. HFD feeding continued for another week.

After having established the survival-enhancing action of NTM peptides in hyperlipidemic (Fig. 1D) and normolipidemic (Fig. S3) animals, we explored the transcriptional mechanism of hyperlipidemic hypersensitivity to LPS. To this end, we tested pathway-selective inhibitors of the nuclear transport checkpoint in the high fat diet-fed *ldlr*^*−/−*^ mice using two in vivo protocols comprising the long- and short-term treatments (Fig. 3).

**Figure 4:**
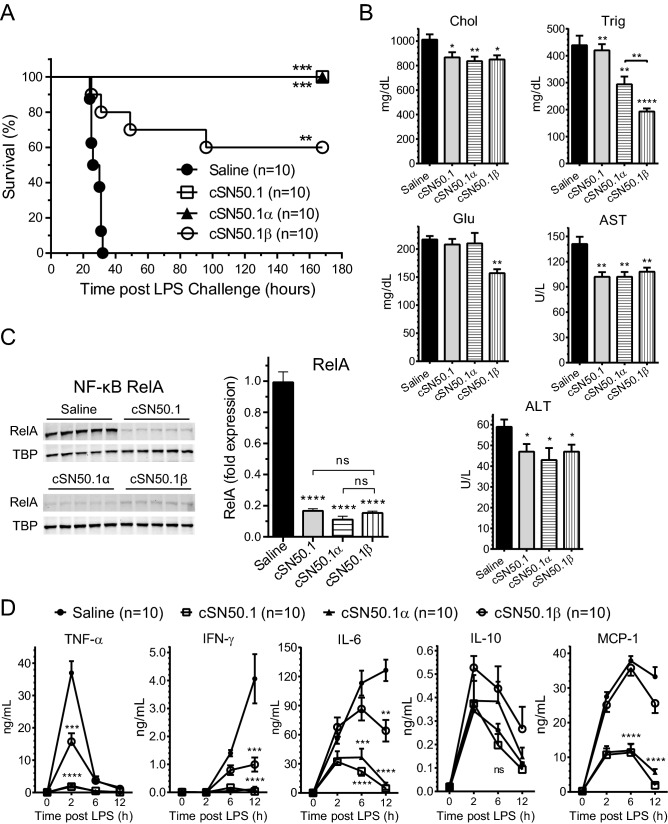
HFD-induced hypersensitivity to lethal microbial inflammation induced by LPS is suppressed by targeting nuclear import pathways of SRTFs and MTFs. *ldlr*^*−/−*^ female mice were treated according to the *long-term* *treatment* *protocol* (see Fig. 3A). Briefly, 15-week-old mice fed with HFD and treated BID with saline, biselective NTM, cSN50.1 peptide, or monoselective NTMs, cSN50.1α or cSN50.1β peptides for three weeks were challenged with non-lethal dose of LPS (10 μg/g) and observed for 7 days. (**A**) All mice treated with biselective, and Imp α5-selective NTMs were protected from death caused by LPS shock. Treatment with Imp β1-selective NTMs resulted in 60% survival. Data is presented as Kaplan–Meier survival plot with *p* value calculated by log rank analysis, **p < 0.005, ***p < 0.0005. (**B**) Levels of metabolic markers (cholesterol, triglycerides, glucose) and liver transaminases (AST, ALT) were measured in blood plasma collected at the end of week 3 of HFD feeding and NTM treatment, before LPS challenge. (**C**) Nuclear translocation of NF-κB RelA, was determined by quantitative immunoblotting of liver nuclear extracts obtained at the time of sacrifice. Western blots were analyzed using LI-COR Odyssey infrared imaging system (unedited full-length immunoblots are presented in Supplementary Figure S5A). Data in (**B**,**C**) is presented as a mean ± SEM (n = 10). Statistical significance was determined by ordinary One-way ANOVA with Holm–Sidak test for multiple comparison, *p < 0.05, **p < 0.005, ****p < 0.0001. (**D**) Cytokines (TNF-α, IFN-γ, IL-6, and IL-10) and chemokine MCP-1, levels were determined in blood plasma collected from saphenous vein before and 2, 6, and 12 h after LPS challenge. Data is presented as a mean ± SEM (n = 10). Statistical significance was determined by repeated measures two-way ANOVA analysis of variance with Holm-Sidak test for multiple comparison, **p < 0.005, ***p < 0.0005, ****p < 0.0001.

In the Long-Term Treatment protocol (Fig. 3A), *ldlr*^*−/−*^ mice fed HFD for 3 weeks were simultaneously treated with NTM peptides while control animals received saline (diluent control). All control mice died of LPS shock within 40 h (Fig. 4A). In striking contrast, 100% mice treated with the Imp α5-selective NTM peptide, cSN50.1α, survived along with the mice treated with the biselective NTM, cSN50.1 peptide. This indicates that the Imp α5-mediated signaling pathway to the nucleus contributes to death in LPS-induced microbial inflammation superimposed on preexisting metabolic inflammation caused by HFD.

Importantly, 60% survival was also observed in HFD-fed *ldlr*^*−/−*^ mice treated with Imp β1-selective NTM, cSN50.1β peptide for 3 weeks. These results indicate that the Imp β1-mediated signaling pathway to the nucleus also contributes to death in LPS-induced microbial inflammation superimposed on preexisting metabolic inflammation caused by HFD. Consequently, targeting Imp β1-mediated transport of SREBPs^21^ should improve metabolic markers in the HFD-fed *ldlr*^*−/−*^ mice.

### Treatment with nuclear import pathway-selective NTMs improves metabolic markers in the blood and reduces nuclear content of proinflammatory transcription factor, NF-κB RelA, in the liver

Indeed, hypertriglyceridemia was precipitously reduced by the Imp β1-selective NTM, cSN50.1β peptide, producing a 2.5-fold decline in blood triglycerides after a 3-week treatment. Notably, this new monoselective inhibitor of the Imp β1-mediated signaling pathway also reduced the elevated blood glucose level (Fig. 4B). Hypercholesterolemia was significantly albeit moderately lowered by the biselective and two monoselective NTM peptides in hyperlipidemic mice before their challenge with the inducer of microbial inflammation, LPS. Liver transaminases (AST and ALT) were also moderately reduced. Altogether, selective targeting of Imp β1 improves metabolic profile of HFD-fed mice after the 3-week treatment.

We focused further mechanistic analysis of hyperlipidemic hypersensitivity to LPS on the nuclear translocation of the main proinflammatory SRTFs, NF-κB RelA, in the liver cells. The liver nuclear extracts of control hyperlipidemic *ldlr*^*−/−*^ mice treated with saline displayed abundant content of this prominent proinflammatory transcription factor (Fig. 4C). In contrast, the 3-week treatment with biselective or two monoselective NTM peptides significantly reduced the nuclear transport of NF-κB RelA (Fig. 4C). The paucity of the NF-κB RelA in the liver nuclear extracts of animals treated with Imp β1-selective NTM, cSN50.1β peptide was striking. This presumably paradoxical result (cSN50.1β peptide is not targeting Imp α5-mediated nuclear transport of NF-κB RelA) (see Fig. 2A) offers a proof for the activation of the NF-κB RelA signaling pathway by elevated neutral lipids in the liver cells. When accumulation of these noxious metabolites is prevented by the 3-week treatment with Imp β1-selective NTM, the NF-κB RelA is not mobilized. Thus, the very low levels of nuclear NF-κB RelA, the transcriptional vanguard of inflammation in the liver cells’ nuclei, coincided with the effectiveness of the life-saving treatment with biselective and nuclear import pathway-selective NTM peptides.

### Suppression of inflammatory cytokines and chemokines, fatty liver, glycogen depletion, microvascular endothelial injury, and trafficking of neutrophils by differential action of nuclear import pathway-selective NTM peptides

Consistent with the survival data, LPS-induced burst of proinflammatory cytokines [TNF-α, Interferon (IFN)-γ, Interleukin (IL)-6, and chemokine MCP-1] was suppressed by the long-term treatment protocol (3-week) with the biselective and the Imp α5-selective NTM peptides (Fig. 4D). In agreement with the prior studies, the blood level of anti-inflammatory cytokine, IL-10, remained elevated in all treatment groups^33^. The IL-10, also known as cytokine synthesis inhibitory factor (CSIF), has a potent anti-inflammatory properties contributing to amelioration of tissue damage by suppressing activity of immune cells such as Th1 cells, NK cells or macrophages^34^.

The 3-week treatment with the Imp β1-selective NTM, cSN50.1β peptide, only partially suppressed LPS-induced production of TNF-α, IFN-γ, and IL-6. LPS-induced expression of chemokine MCP-1 was not affected by this monoselective NTM.

**Figure 5:**
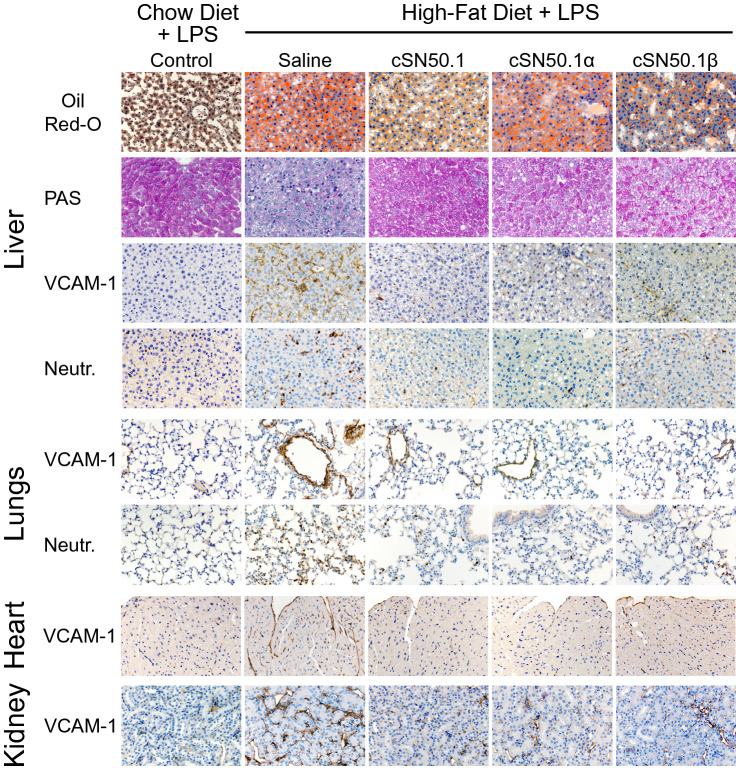
NTMs targeting of nuclear import pathways of SRTFs and MTFs attenuates multi-organ injury in hyperlipidemic mice caused by LPS-induced microbial inflammation. Targeting both nuclear import pathways mediated by Imp α5 and Imp β1 resulted in suppression of LPS-induced glycogenolysis (PAS) in the liver, microvascular endothelial injury manifested by increased expression of VCAM-1; in the liver, lungs, heart, and kidney, and inflammatory cells activation and trafficking (neutrophils in the liver, and lungs). However, accumulation of neutral lipids in the liver (Oil Red O, ORO) was reduced in the mice treated with biselective NTM, cSN50.1 peptide, and monoselective NTM, cSN50.1β peptide, targeting Imp β1-mediated nuclear import of SREBPs. Representative images (×40 magnification) of liver, lungs, heart, spleen and kidney sections in LPS-challenged 15-week-old *ldlr*^*−/−*^ female mice fed chow diet (control) or mice fed HFD and treated BID with saline or with indicated NTMs for three weeks before LPS challenge. Data presented in this figure represents 2 independent experiments completed with 5 mice per treatment group. See “Methods” for details.

The remarkable immunometabolic improvements due to the modulation of nuclear import pathways for SRTFs and MTFs were reaffirmed by an immunocytochemistry analysis of the liver and other organs (Fig. 5). The accumulation of liver neutral lipids stained with Oil-Red-O (ORO)^35^ was reduced by both the biselective NTM, cSN50.1, and the Imp β1-selective NTM, cSN50.1β, peptides. The latter result supports our mechanistic analysis of nuclear transport regarding the paucity of nuclear translocation of the NF-κB RelA in the liver cells of animals treated with the Imp β1-selective NTM (see above). Glycogen breakdown in the liver is detrimental in bacterial and viral infections^36^. LPS-induced glycogenolysis is linked to the hyperglycemia and hypertriglyceridemia mediated by the nuclear import of ChREBP transcription factors involved in the development of MetS^32^. Indeed, we demonstrated an almost total depletion of the glycogen stained with PAS in the saline-treated control mice. This LPS-induced loss of liver glycogen was prevented by the biselective NTM, cSN50.1 peptide, whereas the Imp α5- and Imp β1-selective NTMs, cSN50.1α and cSN50.1β peptides were partially protective indicating that both nuclear transport pathways contribute to the glycogen breakdown (Fig. 5).

Severe microvascular endothelial injury is the main mechanism of lethal LPS shock, the end stage of LPS-induced microbial inflammation^11^. It was manifested by increased expression of Vascular Cell Adhesion Molecule (VCAM)-1, regulated by SRTFs (e.g., NF-κB and STAT1). VCAM-1 was prominently expressed in the endothelial cells of the liver, lungs, heart, and kidney of control animals fed HFD as compared to those on chow diet after LPS challenge (Fig. 5). In contrast, VCAM-1 expression was dramatically suppressed in cSN50.1- and cSN50.1α-treated groups in the 3-week treatment protocol. The cSN50.1β-treated group shows a diminished albeit not completely suppressed expression of VCAM-1.

The activation and trafficking of neutrophils and other myeloid cells into the organs’ parenchyma, such as seen in the liver and lungs, is also associated with microvascular endothelial injury^37^. These inflammatory cells contribute to the oxidant stress^11^. Their trafficking was suppressed in animals treated with biselective, and Imp α5-selective NTMs, cSN50.1 and cSN50.1α peptides (Fig. 5). Notably, targeting Imp β1-mediated nuclear import of SREBPs by cSN50.1β peptide also resulted in a significant reduction of neutrophils migration, consistent with increased survival data (see Fig. 4A). Thus, it is apparent that a significant gain in survival of LPS-challenged hyperlipidemic mice, following a 3-week treatment with the pathway- selective NTM peptides, was linked to their anti-inflammatory and cytoprotective action in the liver and other organs.

**Figure 6:**
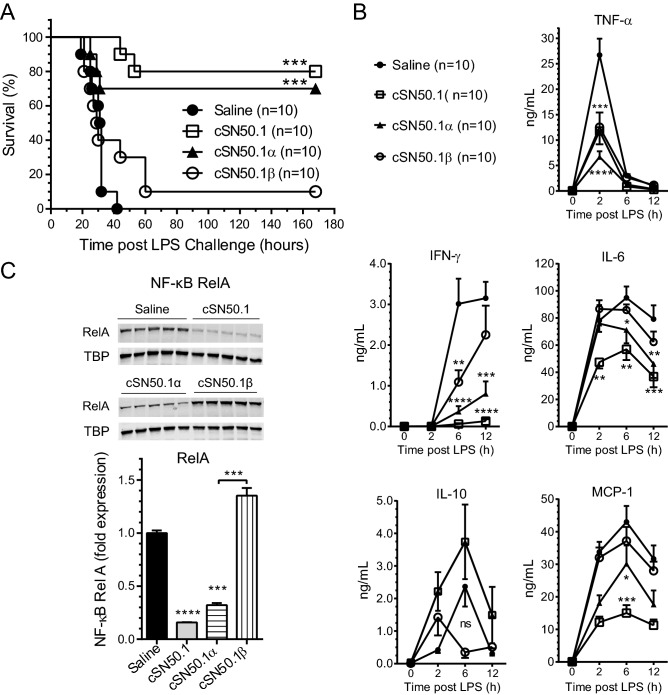
Short-term treatment of LPS-induced lethal microbial inflammation in hyperlipidemic mice with biselective and Imp α5-selective NTMs is effective while Imp β1-selective NTM is ineffective. *ldlr*^*−/−*^ female mice were treated according to the *short-term* *treatment* *protocol* (see Fig. 3B). Briefly, 15-week-old female mice fed with HFD for three weeks were challenged with non-lethal dose of LPS (IP, 10 μg/g in 0.2 mL saline) followed by treatment with 15 doses of saline (control; IP, 0.2 mL), biselective NTM, cSN50.1 peptide, or monoselective NTMs, cSN50.1α or cSN50.1β peptides (33 μg/g/injection in 0.2 mL saline) administered 30 min before LPS challenge and over 3 days thereafter. (**A**) Mice treated with biselective, and Imp α5-selective NTMs display 80% and 70% survival, respectively. Treatment with Imp β1-selective NTMs did not protect the majority of hyperlipidemic mice from death caused by LPS-induced microbial inflammation (10% survival). Data is presented as Kaplan–Meier survival plot with *p* value calculated by log rank analysis, **p < 0.005, ***p < 0.0005. (**B**) Cytokines (TNF-α, IFN-γ, IL-6, and IL-10) and chemokine MCP-1, levels were determined in blood plasma collected from saphenous vein of animals before and 2, 6, and 12 h after LPS challenge. Data is presented as a mean ± SEM (n = 10). Statistical significance was determined by repeated measures two-way ANOVA analysis of variance with Holm-Sidak test for multiple comparison, *p < 0.05, **p < 0.005, ***p < 0.0005, ****p < 0.0001. (**C**) Nuclear translocation of SRTFs NF-κB RelA was determined by quantitative immunoblotting of liver nuclear extracts obtained at the time of sacrifice. Western blots were analyzed using LI-COR Odyssey Infrared Imaging System (unedited full-length immunoblots are presented in Supplementary Figure S5B). Data is presented as a mean ± SEM (n = 10). Statistical significance was determined by ordinary one-way ANOVA with Holm–Sidak test for multiple comparison, ***p < 0.0005, ****p < 0.0001.

Contrariwise, the short-term treatment (see Fig. 3B), with Imp β1-monoselective NTM peptide (see Fig. S2C) administered shortly before the LPS challenge and 3 days thereafter, during the acute stage of LPS shock was largely ineffective (10% survival) indicating that this lipid-lowering treatment requires more chronic regimen (Fig. 6A).

In striking contrast to the ineffective action of Imp β1-monoselective NTM peptide in the short-term protocol, 80% mice acutely treated with the biselective NTM peptide, cSN50.1, survived. Likewise, 70% mice treated with the Imp α5-selective NTM, cSN50.1α peptide, were protected from death (Fig. 6A). The analysis of blood cytokines and chemokines showed the partial suppression of TNF-α, IFN-γ, IL-6, and MCP-1 by the biselective and Imp α5-selective peptides (Fig. 6B) consistent with inhibition of the nuclear import of proinflammatory SRTF, NF-κB RelA, in the liver cells by cSN50.1 and cSN50.1α peptides. They target Imp α5-mediated nuclear import pathway of SRTFs and ChREBP (Fig. 6C).

**Figure 7:**
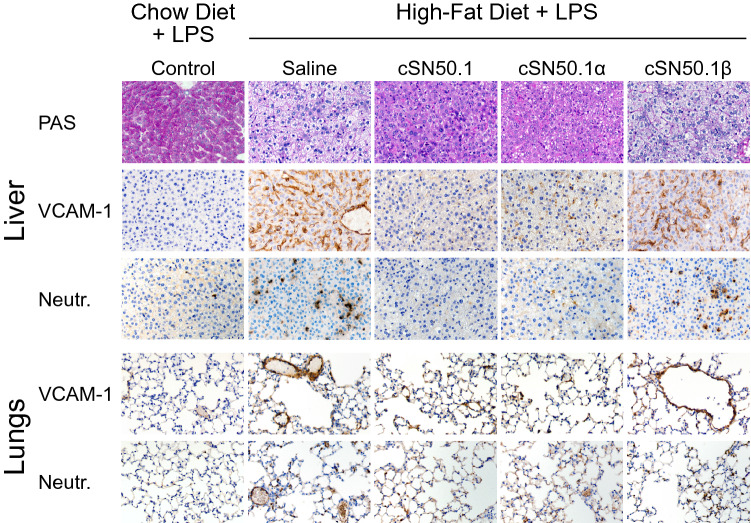
Multi-organ injury in the LPS-induced microbial inflammation in hyperlipidemic mice is prevented only by treatment with NTMs targeting nuclear import pathways of SRTFs and ChREBPs mediated by Imp α5 pathway whereas targeting Imp β1-mediated pathway with cSN50.1β peptide is ineffective in the Short-Term treatment protocol. LPS-induced glycogenolysis (PAS) in the liver, microvascular endothelial injury (VCAM-1; in the liver, and lungs) and inflammatory cells infiltration (neutrophils; in the liver, and lungs) are attenuated by treatment targeting nuclear import pathway mediated by Imp α5 with monoselective NTM cSN50.1α and biselective NTM cSN50.1. Targeting Imp β1-mediated pathway with cSN50.1β peptide is ineffective. Representative images (×40 magnification) of liver and lungs sections in LPS-challenged 15-week-old *ldlr*^*−/−*^ female mice fed chow diet (control) or mice fed HFD and treated with 15 doses of saline or indicated NTMs given 30 min before LPS challenge and three days thereafter. Data presented in this figure represents 2 independent experiments completed with 5 mice per condition group. See “Methods” for details.

Only animals treated with NTMs targeting Imp α5-mediated nuclear import, i.e. cSN50.1 and cSN50.1α peptides, displayed inhibition of glycogenolysis, suppressed VCAM-1 expression in endothelial cells, and the arrest of neutrophil trafficking in the liver and lungs, In striking contrast, short-term administration of Imp β1-selective NTM, cSN50.1β peptide, was ineffective in protecting organs from devastating action of LPS shock in hyperlipidemic mice (Fig. 7), consistent with the survival data (Fig. 6A).

## Discussion

Cumulatively, these results support the concept that hyperlipidemia, the key component of MetS, increases the susceptibility to lethal microbial inflammation. Further, they demonstrate that selective inhibition of Imp α5-mediated nuclear transport of SRTFs and Imp β1-mediated nuclear transport of MTFs counteracts lethal microbial inflammation in hyperlipidemic animals. The nuclear transport checkpoint is an attractive target for therapeutic intervention in this context because NTM peptides administered parenterally significantly offset the lethal microbial inflammation. Imp α5-mediated nuclear import plays a key role in hyperlipidemic hypersensitivity to lethal microbial inflammation since its targeting with the cSN50.1 and cSN50.1α peptides was protective in both long-term and short-term treatment protocols. In contrast, Imp β1-mediated pathway could be effectively suppressed by the long-term treatment with the Imp β1-selective NTM, cSN50.1β peptide whereas short-term administration was not effective. Thus, the transcriptional signaling cascades mediated by importin α5 and importin β1 provide the new nexus for hyperlipidemic hypersensitivity to potentially lethal microbial diseases.

The major step in understanding the mechanism of HFD-induced metabolic inflammation “sensitizing” animals to lethal LPS-induced microbial inflammation is the outcome of the long-term administration of monoselective NTM targeting Imp β1, which ferries SREBPs, during the 3-week feeding HFD. Notably, this long-term protocol was sufficient to abrogate LPS shock in the majority of HFD diet-fed mice. These mice displayed a precipitous decline in blood triglycerides and glucose while the reduction of cholesterol levels was smaller albeit significant. Hence, the genes encoding proteins involved in the synthesis of triglycerides, cholesterol, and glucose were not activated. We reported previously^21^ that biselective NTM, cSN50.1 peptide suppressed the expression of the genes encoding HMG-CoA reductase (a target of statins), ATP citrate lyase, fatty acid synthase, and Nieman-Pick C1-like 1 protein (a key enterohepatic cholesterol absorption receptor), among over 30 genes regulated by SREBPs^38^. Since SREBPs are translocated to the nucleus by Imp β1, its long-term targeting by Imp β1-selective peptide was sufficient to protect 60% of the *ldlr*^*−/−*^ mice fed HFD from lethal microbial inflammation.

Paradoxically, we discovered the striking paucity of NF-κB RelA in the nuclei of animals fed HFD and treated for 3 weeks with Imp β1-selective peptide (Fig. 4C). How the long-term treatment with Imp β1-selective peptide suppresses nuclear translocation of NF-κB RelA, the master proinflammatory SRTF? As we documented above, the Imp β1-selective peptide does not directly inhibit the nuclear translocation of NF-κB RelA (see Fig. 2A). Rather this new selective inhibitor of nuclear transport checkpoint prevented the accumulation of cholesterol, triglycerides, and fatty acids in the liver cells (see Oil-Red-O staining of neutral lipids in the liver cells in Fig. 5). Hence, their activation of the main proinflammatory signaling pathway mediated by NF-κB RelA was averted. Inhibition of SREBPs nuclear import during the long-term (3 weeks) treatment protocol (see Fig. 3A) counteracted the metabolic derangements caused by the HFD thereby warding off signs of steatohepatitis and hyperlipidemic hypersensitivity to LPS shock. Thus, the long-term treatment of HFD-induced hyperlipidemia and hyperglycemia with the Imp β1-selective, cSN50.1β peptide offers a promising strategy to prevent fatty liver and subsequent steatohepatitis thereby reducing the susceptibility to the end-stage microbial inflammation such as LPS shock.

In contrast, the short-term (“just-in-time”) treatment protocol (see Fig. 3B), with the importin β1-selective NTM, administered coincidently with the LPS challenge, was not effective in reducing the lethal outcome (see Fig. 6A). We infer that, SREBPs do not seem to contribute to the LPS shock in the acute phase of microbial inflammation.

The discovery of the nuclear transport pathway mediated by importin α5 for the Carbohydrate-Responsive Element-Binding Proteins (ChREBP) family^39^, is of particular significance. It explains why selective targeting of the importin α5-mediated pathway prevented glycogen depletion observed in the livers of hyperlipidemic animals succumbing to the LPS shock. Hence, the resulting hyperglycemia and hypertriglyceridemia were reduced. This metabolic duo comprises “the deadly combination” underlying MetS^40^. As ChREBPs, the regulators of glucose homeostasis, are translocated to the nucleus through the same pathway as proinflammatory SRTFs, targeting the Imp α5-mediated pathway by selective NTM peptide (cSN50.1α) was equally protective in comparison to the biselective NTM in hyperlipidemia-aggravated LPS shock. We submit that hyperlipidemic hypersensitivity to LPS shock is chiefly mediated by the proinflammatory SRTFs along with ChREBPs in the short-term treatment protocol. The documented outcome of inhibiting ChREBPs access to the nucleus in our study, is in agreement with previous reports showing that ChREBPs deficiency results in glycogen synthesis while triglyceride formation is reduced^32^. Another evidence for the ChREBPs involvement in metabolic syndrome, is their cooperation with SREBP1c, in induction of glycolytic and lipogenic genes^32,41^. The nuclear transport checkpoint inhibition of importin α5 and importin β1 would keep these genes silent.

**Figure 8:**
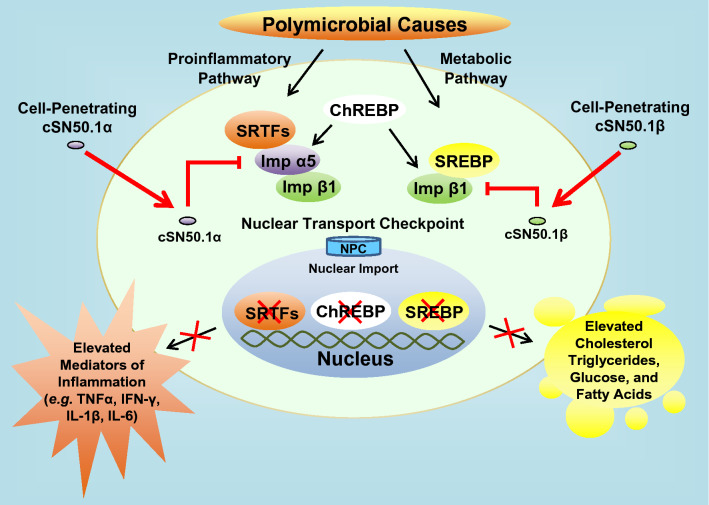
Microbial inflammation is interwoven with metabolic inflammation. Polymicrobial causes (viruses, bacteria, fungi, and protozoa) evoke two signaling pathways (proinflammatory and metabolic). These pathways can be independently targeted by new, pathway selective NTMs, cSN50.1α and cSN50.1β, that respectively bind nuclear transport shuttles, Imp α5 and Imp β1, to modulate Imp α5-mediated nuclear import of stress responsive transcription factors (SRTFs), and Imp β1-mediated nuclear import of metabolic transcription factors (MTFs) such as SREBPs. Transcription factors ChREBPs can be imported to the nucleus either by heterodimeric complex Imp α5/Imp β1 or by Imp β1 alone. Inhibition of nuclear transport of SRTFs reduces expression of genes encoding mediators of inflammation, cytokines, chemokines, signal transducers, and cell adhesion molecules (see Fig. S1). Inhibition of ChREBPs, that regulate genes encoding proteins involved in glucose homeostasis, reduces hyperglycemia and hypertriglyceridemia, inhibition of nuclear transport of SREBPs reduces expression of genes encoding proteins involved in synthesis of cholesterol, triglycerides, and fatty acids. The accumulation of these metabolites can activate proinflammatory pathway mediated by NF-κB RelA and other SRTFs thereby aggravating microbial inflammation (see text for details; *NPS* nuclear pore complex).

As depicted in Fig. 8, different causes of microbial diseases (bacteria, viruses, fungi, protozoa) activate in immune and non-immune cells the proinflammatory signaling pathway mediated by transcription factors, SRTFs. In hyperlipidemic and hyperglycemic host with MetS, the metabolic transcription factors, ChREBPs, and SREBPs, are mobilized. The products of the genes regulated by these transcription factors (cholesterol, triglycerides, glucose) also activate proinflammatory signaling pathway mediated by the Imp α5, thereby producing more severe and difficult to control stage of microbial inflammation. This advanced stage of microbial inflammation comprises acute respiratory distress syndrome (ARDS), septic cardiomyopathy, microvascular thrombosis (known as Disseminated Intravascular Coagulation), and Acute Kidney Injury. These life-threatening disorders result from the microvascular endothelial injury due to genomic storm that underlies septic shock, the end stage of microbial inflammation^10^.

The proinflammatory and metabolic pathways involved in the end stage of lethal microbial inflammation depend on the nuclear transport shuttles, importins α5 and β1. The pathway-selective cell-penetrating NTM peptides control the nuclear transport checkpoint staffed by these importins. The NTM peptides modulate the cross-talk between metabolic and microbial signaling pathways thereby suppressing the potentially lethal inflammatory response to infections aggravated by hyperlipidemia and hyperglycemia. The NTM peptides also enhance the innate immunity-mediated clearance of bacteria in a polymicrobial septic shock model^37^. Thus, targeting of the nuclear transport checkpoint offers a new and potentially effective countermeasure (as an adjunct to the pathogen-specific antimicrobial therapies) for microbial diseases in a host compromised by underlying metabolic syndrome. Our study explains the transcriptional mechanism of hyperlipidemic hypersensitivity to lethal microbial inflammation caused by microbial agents. We provide new experimental evidence that the immunometabolic axis mediated by importin α5 and importin β1 underlies hyperlipidemic hypersensitivity to lethal microbial inflammation. This immunometabolic axis can be dismantled by selective targeting of the nuclear transport checkpoint.

In summary, we successfully enabled two selective inhibitors of nuclear transport to dissect the transcriptional mechanism of hyperlipidemic hypersensitivity to microbial inflammation. Our findings are of significant relevance to individuals displaying signs of metabolic syndrome that predisposes them to life-threatening microbial diseases, including recent outbreaks of COVID-19^6–9^ as well as autoimmune and allergic disorders^3,42^. The biselective NTM peptide (AMTX-100 CF) is undergoing Phase I/II treatment trial for inflammatory skin diseases (NCT04313400).

## Methods

### Synthesis and purification of cell-penetrating nuclear transport modifier peptides

Cell-penetrating NTM peptides, cSN50.1 (AAVALLPAVLLALLAPCVQRKRQKLMPC, 2986 Da), cSN50.1α (AAVALLPAVLLAVLAPCVQRKRQKLMPC, 2972 Da), cSN50.1β (AAVALLPAVLLALLAPCVQRDEQKLMPC, 2946 Da), and NULL cSN50.1 (AAVALLPAVLLAVLACVQRDEQKLMPC, 2932 Da) were synthesized as described elsewhere^21,26,33,43^. Briefly, the peptide chain was assembled through the Solid Phase Peptide Synthesis (SPPS) according to standard Fmoc chemistry protocols using an automated peptide synthesizer FOCUS XC (AAPPTec, Louisville, KY). Crude peptides were removed from resin with a TFA cleavage cocktail and purified by dialysis against double-distilled water in 1 kDa membrane (Spectra/Por 7; Spectrum Laboratories, Rancho Dominguez, CA). Purity and structure of final products were verified by analytical C18 RP HPLC (Beckman Coulter GOLD System, Brea, CA) and MALDI mass spectroscopy (Voyager Elite; PerSeptive Biosystems, Framingham, MA).

### Nuclear translocation of SRTFs and MTFs in cultured cells

#### Murine macrophage RAW 264

RAW cells (ATCC) were cultured according to the supplier’s instruction in 10 cm dishes until confluent. Cells were pretreated with NTM peptides (30 µM cSN50.1, cSN50.1α or cSN50.1β) 30 min before LPS stimulation (10 ng/mL; *E.* *coli* O127:B8; Sigma). Cells were incubated for 6 h at 37 °C in 5% CO_2_, then harvested, lysed with hypotonic buffer^37^ containing 2% NP-40 and washed 3 times to yield clean nuclei. Nuclear proteins were obtained by high-salt extraction (450 mM NaCl; 4 °C, 2000 rpm 30 min). LPS-stimulated cells not-treated with NTM peptides and unstimulated cells served as positive and negative controls, respectively.

#### Human embryonic kidney 293T

HEK 293T cells (ATCC) were cultured according to the supplier’s instruction in 10 cm dishes until confluent. Cells were pretreated for 15 min with 1% hydroxypropyl-beta-cyclodextrin (HPCD) in DMEM containing 1% delipidated serum (DLS) to lower the cell content of sterols. HPCD was removed and cells were treated with 30 µM cSN50.1, cSN50.1α or cSN50.1β in DMEM containing 5% DLS for 2 h (see^21^ for details) HPCD-modified cells not treated with NTM peptides and unmodified cells served as positive and negative controls, respectively. Nuclear extracts were obtained as described above.

#### Human hepatocyte carcinoma

HepG2 cells (ATCC) were cultured according to the supplier’s instruction in 10 cm dishes until confluent. Cells were starved for 24 h in DMEM containing 5.5 mM glucose then refed with 25 mM glucose (HG) with addition of 100 nM insulin (In) and 30 µM cSN50.1, cSN50.1α or cSN50.1β for 24 h. HG/In-stimulated cells not treated with NTM peptides and unstimulated cells served as positive and negative controls, respectively. Nuclear extracts were obtained as described above. Viability of cells used in cell-based assays was greater than or equal to 80%.

The nuclear content of NF-ĸB RelA, pSTAT1 and cFos in RAW cells, SREBP2 in HEK 293T cells, and ChREBP in Hep G2 cells was determined by quantitative immunoblotting using rabbit monoclonal anti-NF-κB p65 (RelA) antibody (Cell Signaling, Cat# 8242), mouse monoclonal anti-phospho STAT1 pY 701 antibody (BD Bioscience, Cat# 612132), mouse monoclonal anti-cFos antibody (Santa Cruz, Cat# sc-166940), rabbit polyclonal anti-SREBP2 antibody (Invitrogen, Cat# PA5-88943), rabbit polyclonal anti-ChREB antibody (Novus Biologicals, Cat# NB400-135), respectively. Rabbit polyclonal anti-Histone 3 antibody (Cell Signaling, Cat# 9715) was used to measure Histone 3 as a nuclear loading control for normalization. Immunoblots were analyzed on a LI-COR Odyssey Infrared Imaging System. Each cell-based experiment was performed in duplicates or triplicates and repeated at least twice to assure experimental significance and reproducibility.

### Animal studies

Animal experiments were carried out in compliance with the ARRIVE guidelines and in strict accordance with the Guide for the Care and Use of Laboratory Animals of the National Institutes of Health, and submitted protocols were approved by the Vanderbilt University Institutional Animal Care and Use Ethics Committee (Permit Number: A3227-01). Mice were closely monitored during the course of experiments and euthanized by Isoflurane inhalation followed by cervical dislocation upon expression of the signs of moribund state. Survivors were euthanized at the experimental end point.

#### Mouse model of endotoxin shock

We used this model to compare biselective and monoselective NTM peptides with the control “loss of function” Null cSN50.1 peptide (see Fig. S3). 15-week-old female C57BL/6 mice (The Jackson Laboratory, 20 g body weight) selected into five experimental groups (5 mice/group) using double blinded randomization method. Briefly, mice ear tag’s numbers were written down on 25 pieces of paper, which were folded, placed in a receptacle, and shaken. Similarly, another 25 pieces of paper were marked with the numbers corresponding to the treatment groups, five of each [1——vehicle (saline); 2—cSN50.1; 3—cSN50.1α; 4—cSN50.1β, 5—Null cSN50.1]. Paper was folded, placed in a separate receptacle and well shaken. Selection was completed by drawing ear tag numbers and pairing them with the treatment group number drawn from second receptacle. Each experiment was performed twice to assure statistical significance and experimental reproducibility. Mice were challenged by IP injection of lethal dose of LPS (700 µg in 200 µL saline; E. coli strain O127:B8 from Sigma) and treated intraperitoneally (IP) with 7 doses of vehicle (saline 200 μL/dose), cSN50.1, cSN50.1α, cSN50.1β or Null cSN50.1 peptides (all at 33 mg/kg/dose) reconstituted in 200 μL of water-saline solution (1:1, v/v). The treatment was conducted in the following regimen: 30 min before and 30, 90, 150, 210, 360, and 720 min after LPS challenge. Blood samples (~ 50 µL) were collected from the saphenous vein in EDTA-coated tubes (Sarstedt) before and at 2, 6, 12 and 24 h post LPS challenge. Survivors were euthanized by overdosed inhalation of isoflurane following cervical dislocation at 72 h post LPS challenge.

#### Mouse model of hyperlipidemia-induced susceptibility to microbial inflammation

An 8-week-old *ldlr*^*−/−*^ female mice (B6.129S7-Ldlr^tm1Her^/J on C57BL/6J genetic background; The Jackson Laboratory) were placed on nonirradiated regular chow diet for 4 weeks. Four experimental groups, vehicle, cSN50.1, cSN50.1α and cSN50.1β (5 mice/group) were selected using double blinded randomization method described above. We used saline as a control representing vehicle for all NTM peptides used in this study. This type of control is used in the FDA-approved clinical studies of human subjects in which tested new drug (including therapeutic peptides) is administered. Each experiment was performed twice to assure statistical significance and experimental reproducibility. Metabolic challenge was instituted in 12-week-old *ldlr*^*−/−*^ female mice fed a high-fat, high-cholesterol diet (HFD) for three weeks before LPS (10 μg/g, *E.* *Coli* O127:B8; Sigma) induction of microbial inflammation. Survivors were fed HFD for another week until the end of experiment. Body weight and food consumption was monitored weekly. Blood samples (~ 50 µL) were collected from the saphenous vein of 6-h-fasting mice in EDTA-coated tubes (Sarstedt) before HFD, before LPS administration and at 2, 6, and 12 h after LPS injection.

##### Long-term NTM treatment protocol (see Fig. 3A)

Mice were treated with the intraperitoneal injection (IP) of NTM peptides, biselective cSN50.1, and monoselective cSN50.1α and cSN50.1β (33 μg/g/injection in 200 μL saline) two times a day (BID) for the course of HFD feeding (3 weeks before LPS challenge and 1 week thereafter). Control group of animals received intraperitoneal injection of saline (200 μL). Survivors were euthanized by overdosed inhalation of isoflurane following cervical dislocation at 168 h post LPS challenge.

##### Short-term NTM treatment protocol (see Fig. 3B)

In this protocol, NTM peptides (biselective cSN50.1, or monoselective cSN50.1α or cSN50.1β; 33 μg/g/injection in 200 μL saline) or vehicle (200 μL saline) were administered through IP injections at 30 min before and 0.5, 1.5, 2.5, 3.5, 6, 9, 12, 18, 24, 30, 36, 42, 48 and 60 h after LPS challenge. Survivors were euthanized by overdosed inhalation of isoflurane following cervical dislocation at 168 h post LPS challenge.

### Preparation of nuclear extracts from the liver cells

Nuclear extracts were prepared from frozen livers as previously described^21^. Briefly, liver pieces were disrupted in a Dounce hand homogenizer on ice in hypotonic lysis buffer^26^ containing 2% NP-40, protease and phosphatase inhibitors (Roche), vortexed, and left on ice for 20 min. Nuclei were pelleted at 10,000×g and washed twice in hypotonic lysis buffer. Nuclear proteins were extracted with high salt solution (450 mM) by shaking nuclei at 2000 rpm for 1 h at 4 °C. Extracts were analyzed by quantitative immunoblotting using rabbit monoclonal anti-NF-κB RelA (p65) antibody (Cell Signaling, Cat# 8242). Mouse monoclonal anti-TATA binding protein antibody (TBP, Abcam, Cat# ab818) was used as nuclear loading control for normalization. Immunoblots were analyzed on a LI-COR Odyssey Infrared Imaging System.

### Histology

Organ samples (liver, kidney, heart, and lungs) were collected and fixed overnight in 10% formalin, routinely processed, embedded in paraffin, sectioned at 5 μm and stained with Hematoxylin and Eosin (H&E) or Periodic Acid- Schiff-hematoxylin to assess injury, hemorrhage, and liver glycogen stores, respectively. Separately, pieces of liver were embedded in OCT buffer and frozen on dry ice. Cryostat sections were stained with Oil-red-O to determine accumulation of neutral lipids. Immunohistochemistry analyses with antibodies against VCAM-1 (Abcam) and neutrophils (Abcam) were performed on the Leica Bond Max following standard protocols in the Translational Pathology Shared Resource at Vanderbilt University Medical Center.

### Cytokine/chemokine and E-selectin assays

Blood plasma levels of cytokines (TNFα, IL-6, IL-10, IFN-γ), chemokine MCP-1, and E-Selectin were determined using Cytometric Bead Array (BD BioSciences) following manufacturer’s protocol and analyzed in the Vanderbilt University Medical Center Flow Cytometry Shared Resource as previously described^33^.

### Statistical analysis

Statistical analysis was performed using tools built-in Prism 6 software (GraphPad). Cytokine (TNFα, IL-6, IL-10, IFN-γ) and chemokine MCP-1 levels in plasma collected from the same animals at different time points were evaluated by repeated measures two-way ANOVA analysis of variance with Holm-Sidak’s post-test for multiple comparison. Survival data were plotted as Kaplan–Meier survival curves and analyzed by the log-rank test. Immunoblots of SRTFs and MTFs in nuclear extracts, and blood chemistry (Chol., Trig., Glu., ALT, AST) from Long-Term NTM Treatment Protocol were analyzed by ordinary One-way ANOVA with Holm-Sidak test for multiple comparison. Blood chemistry (Chol., Trig., Glu., ALT, AST) and E-Selectin levels from Short-Term NTM Treatment Protocol were analyzed by nonparametric *t* test with Mann–Whitney rank comparison. Data are presented as the means ± SEM and *p* values of < 0.05 were considered significant.

## Supplementary Information

Supplementary Information 1.
