# Supplementary material for: Hyperlipidemic hypersensitivity to lethal microbial inflammation and its reversal by selective targeting of nuclear transport shuttles
Source: Sci Rep. 2021 Jun 7;11:11907. doi: 10.1038/s41598-021-91395-w (PMC8184916; doi:10.1038/s41598-021-91395-w)
Supplement: Supplementary file 1 — Supplementary Information 1. [file 41598_2021_91395_MOESM1_ESM.pdf]

SUPPLEMENTARY MATERIALS

Hyperlipidemic Hypersensitivity to Lethal Microbial Inflammation and its Reversal by Selective Targeting of Nuclear Transport Shuttles

Yan Liu[1,2,#], Jozef Zienkiewicz[1,2,#], Kelli L. Boyd[3], Taylor E. Smith[1,2,$], Zhi-Qi Xu[1,2], and Jacek Hawiger[1,2,4,*]

[1] Vanderbilt University School of Medicine, Department of Medicine, Division of Allergy, Pulmonary and Critical Care Medicine, Nashville, Tennessee, United States of America

[2] Department of Veterans Affairs, Tennessee Valley Health Care System, Nashville, Tennessee, United States of America

[3] Vanderbilt University School of Medicine, Department of Pathology, Microbiology and Immunology, Nashville, Tennessee, United States of America

[4] Vanderbilt University School of Medicine, Department of Molecular Physiology and Biophysics, Nashville, Tennessee, United States of America

[#] Y.L. and J.Z. contributed equally to this study.

[$] T.E.S. is pursuing her M.D. degree at the Indiana University School of Medicine, Indianapolis, Indiana, United States of America

* To whom correspondence should be addressed:
Jacek Hawiger
Vanderbilt University Medical Center
21st Avenue South, T-1218, MCN
Nashville, TN 37232, USA
phone: +1 (615) 828-8718
e-mail: jack.hawiger@vumc.org

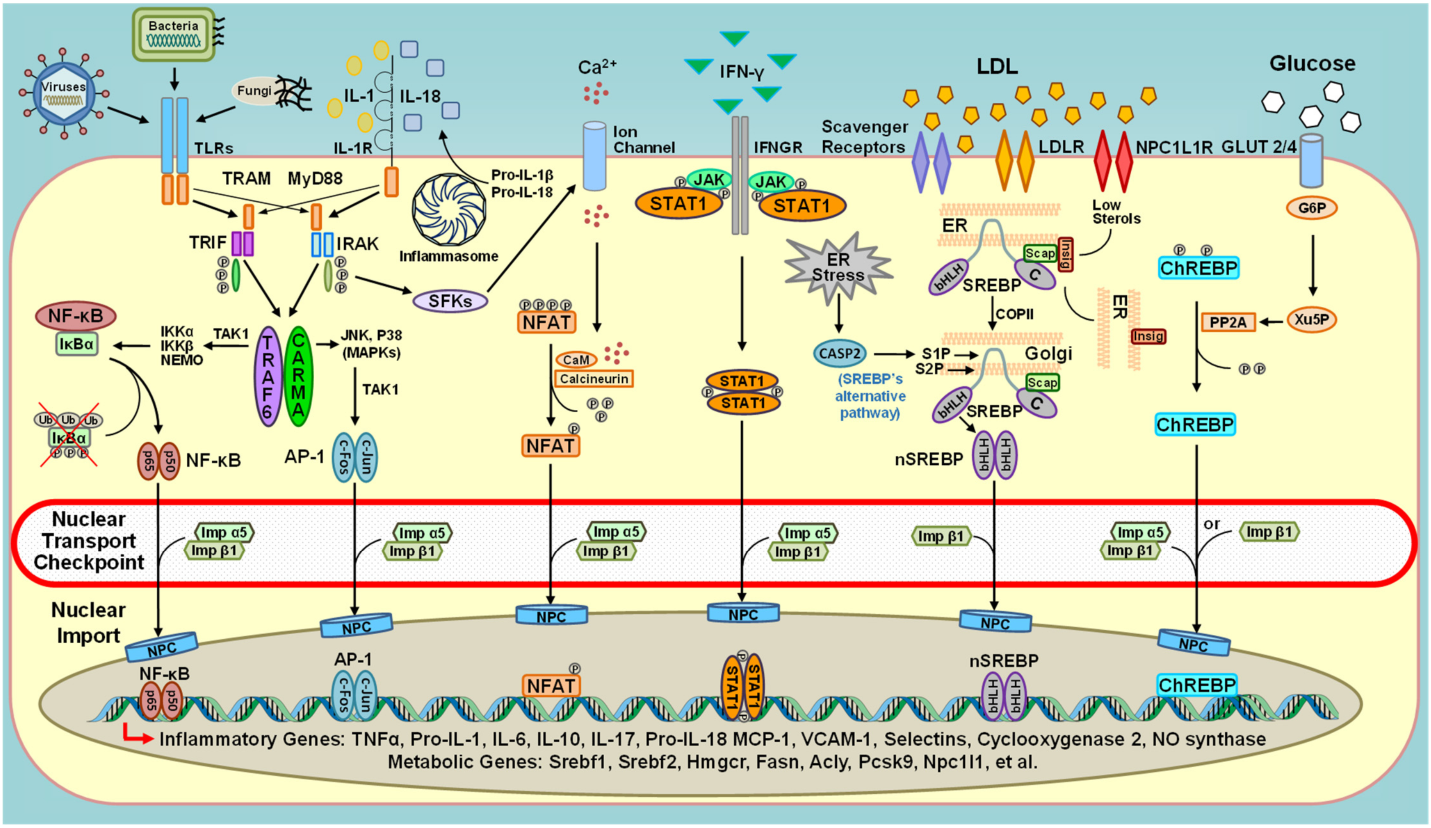

Supplementary Fig. S1. Signaling to the Nucleus by Inflammatory and Metabolic Transcriptional Pathways. In response to multiple proinflammatory cues outside of cell, the six selected transcriptional cascades, depicted schematically in this figure, are activated to trigger signaling to the genome in the cell's nucleus. This signaling is dependent on multiple receptors and their adapters, and other signal transducers (kinases, phosphatases, ubiquitin modifiers, and nuclear import adaptors) that form signalosomes and other multimeric complexes. Proinflammatory Stress-Responsive Transcription Factors (SRTFs) encompass NF-κB, AP-1 (cFos, cJun), NFAT, and STAT1, among others. Metabolic Transcription Factors (MTFs) comprise Sterol Regulatory Element Binding Proteins (SREBPs) and Carbohydrate Regulatory Element Binding Proteins (ChREBPs), among others. Depicted SRTFs and MTFs converge at the Nuclear Transport Checkpoint "staffed" by Importin α5 (Imp α5) and importin β1 (Imp β1), among others. Imp α5 recognizes the Nuclear Localization Sequence (NLS) displayed in depicted SRTFs while Imp β1 recognizes the basic Helix-Loop-Helix leucine-rich zipper (bHLH-Zip) domain displayed in the SREBPs and ChREBPs. Moreover, Imp β1 recognizes its cognate binding site (importin beta binding domain) on Imp α5 upon attachment of its "payload" (i.e. transcription factor) to guide it to the Nuclear Pore Complex (NPC) and the genome. A nuclear form of SREBPs (nSREBPs) is ferried to the cell's nucleus solely by the Imp β1, which recognizes SREBP's N-terminal motif, bHLH-Zip. Other metabolic transcription factors, ChREBPs, display both bHLH-Zip and NLS. Once in the nucleus, SRTFs and MTFs bind to their cognate transcription factor binding sites in the gene promoters and enhancers to initiate transcription of the multiple genes that encode mediators of inflammatory and metabolic pathways. Some of these inflammatory and metabolic genes are displayed in the nucleus. The interaction between "activated" or "processed" TFs and the nuclear transport shuttles can be inhibited by the cell-penetrating peptides termed Nuclear Transport Modifiers (NTMs). Hence, NTMs dismantle the nuclear transport checkpoint by competitive binding to Imp α5 or Imp β1 thereby silencing the inflammatory regulome in the genome (for details: see text and ref.11).

#

Design of Nuclear Transport Modifiers (NTMs)

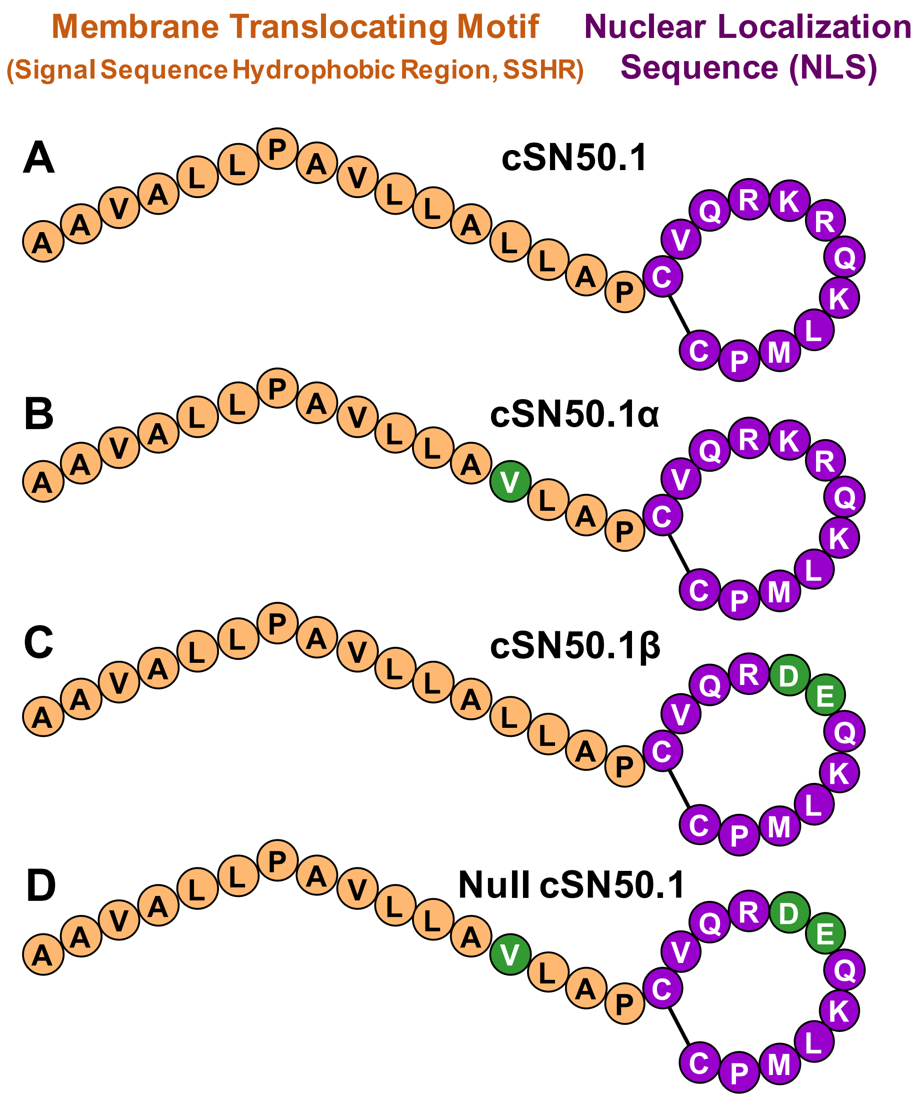

Supplementary Fig. S2. Design of biselective and monoselective NTMs and the inactive NTM peptide as their control. A. The biselective NTM, cSN50.1 peptide. **B.** The Imp α5-selective NTM, cSN50.1α peptide. The L13V substitution in SSHR domain (indicated as green circle) disrupts hydrophobic interaction with importin beta while maintaining the membrane translocating function in cSN50.1α peptide. **C.** The Imp β1-selective NTM, cSN50.1β peptide. The K21D and R22E substitution in the NLS region (marked by the green circles) allows the cSN50.1β peptide to selectively interact with importin β1. **D.** The control NTM, Null CSN50.1 peptide. This NTM has a loss of function substitutions L13V, and K21D/R22E (green circles) and represents an inactive control peptide.

#

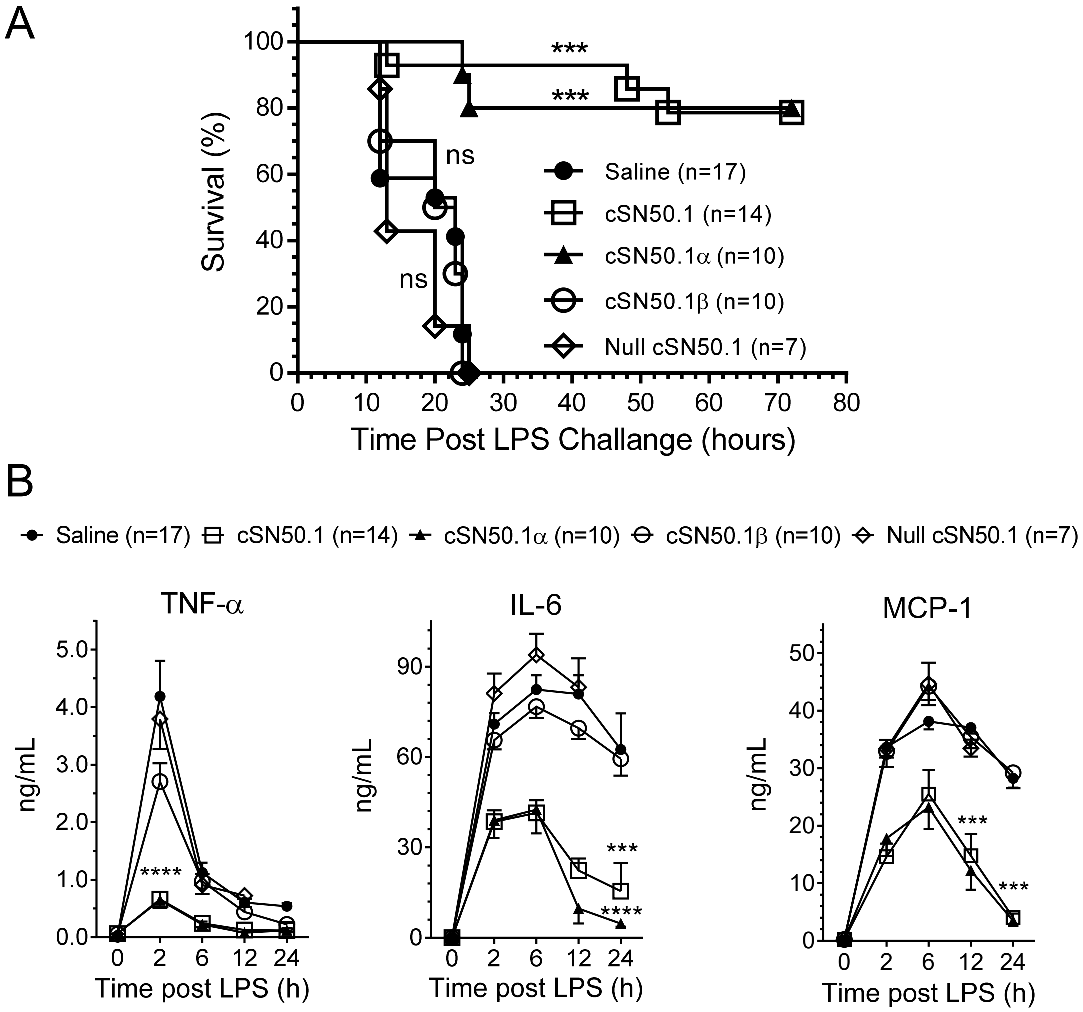

Supplementary Fig. S3. Inactive control peptide does not increase survival nor suppresses production of cytokines and a chemokine in lethal microbial inflammation caused by LPS. . Chow diet-fed C57BL/6 female mice (15-week-old, approx. 20g) received a single dose of LPS (700 µg in 0.2 mL saline) administered through IP injection. LPS-challenged mice were treated by either active NTMs (cSN50.1, cSN50.1α, cSN50.1β) or controls, inactive NTM, Null cSN50.1 peptide or vehicle (saline) also through IP injection according to following treatment regimen: 30 min before and 30, 90, 150, 210, 360, and 720 min after LPS challenge. Blood samples (~50 µL) were collected from the saphenous vein in EDTA-coated tubes (Sarstedt) before and at 2, 6, 12 and 24 h post-LPS challenge. Survivors were euthanized 72 hrs. post LPS challenge. **A.** Survival data presented as Kaplan-Meier plot with p value calculated by log rank analysis, *** $p < 0.0005$. **B.** Cytokines (TNF-α, IL-6) and chemokine MCP-1 levels were determined in blood plasma. Data is presented as a mean ± S.E.M. Statistical significance was determined by repeated measures two-way ANOVA analysis of variance with Holm-Sidak test for multiple comparison, *** $p < 0.0005$, **** $p < 0.0001$.

Supplementary Figure S4. Unedited full-length immunoblots used for preparation of Figure 2.
All SDS PAGE electrophoreses were performed on 4% - 20% gradient gels and proteins content were transferred into membranes using Trans-Blot Turbo System (Bio-Rad). Membranes were then cut along 35kDa line and bottom parts were immunoblotted with rabbit polyclonal anti-Histone 3 primary antibody following by

goat anti-rabbit IRDye 680RD secondary antibodies for detection in 700 nm channel. The upper parts were used for immunoblotting analyses of the protein targets listed in the Methods section following by blotting with donkey IRDye 800CW secondary antibodies for detection in 800 nm channel. After immunoblotting was completed, parts of membrane were placed together on the Odyssey CLx Infrared Imaging System (LI-Cor) and scanned with both channels. Images were quantitatively analyzed using Image Studio 3.1 software (Li-Cor).

Suppl. Fig. S4A. Unedited immunoblot used for Fig. 2A

NF-κB RelA (p65) in nuclear fractions of LPS-stimulated RAW 264 cells

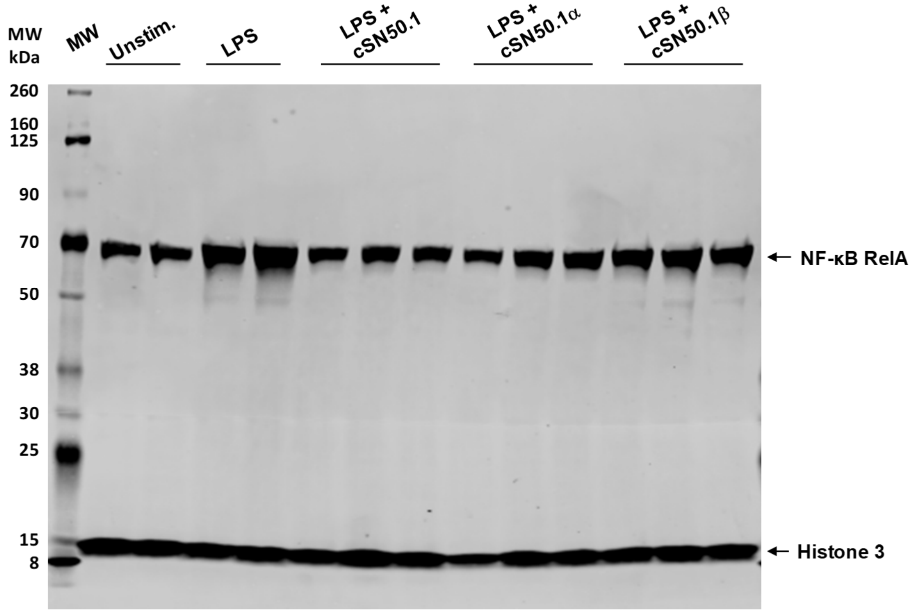

Suppl. Fig. S4B. Unedited immunoblot used for Fig. 2B

Phosphorylated STAT-1 α (91kDa) & β (87kDa) isoforms in nuclear fractions of LPS-stimulated RAW 264 cells (NS – nonspecific bands).

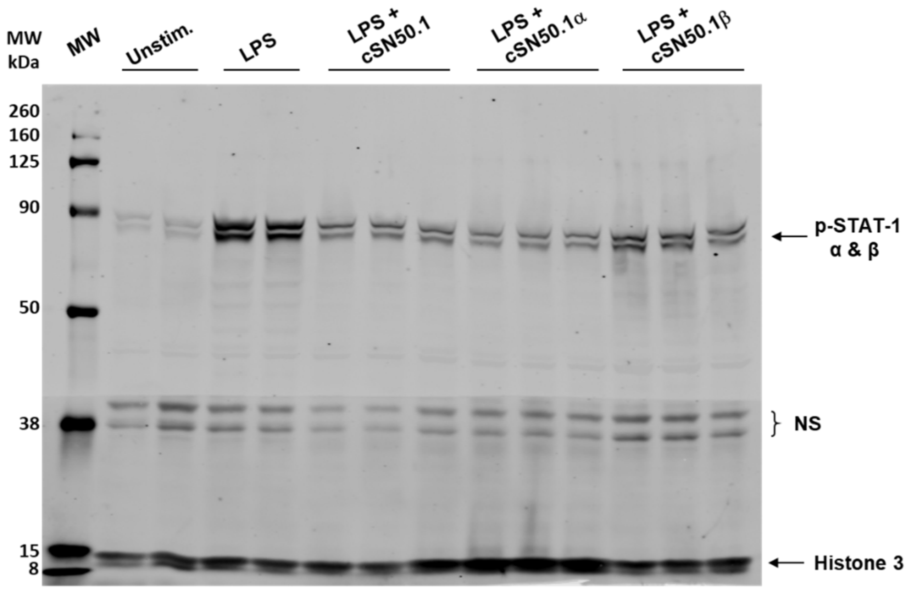

#

Suppl. Fig. S4C. Unedited immunoblot used for Fig. 2C
cFOS (42 kDa) in nuclear fractions of LPS-stimulated RAW264 cells. Due to the limited access to fresh preparation of nuclear fractions, cFos was analized on previously blotted membrane (NF-κB RelA and pSTAT-1) (NS – nonspecific bands).

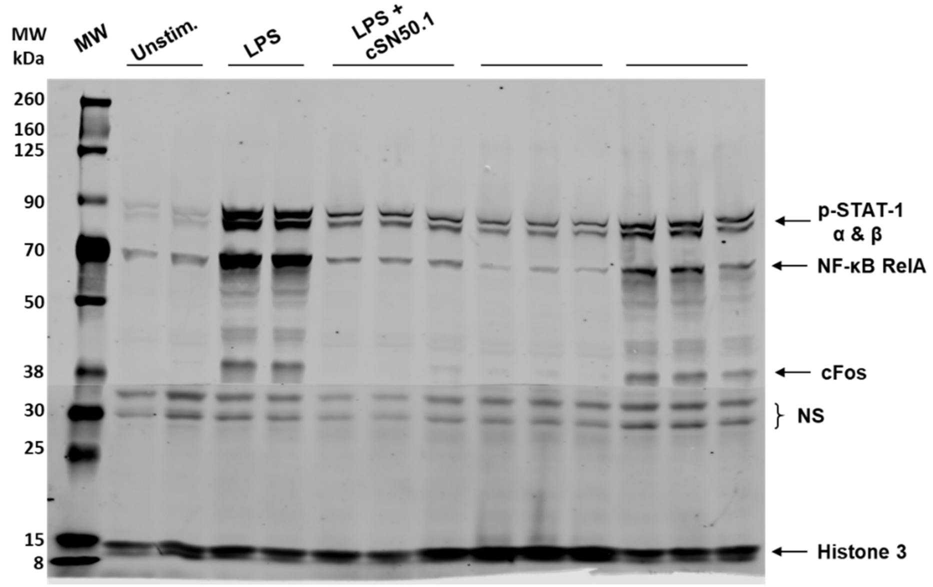

Suppl. Fig. S4D. Unedited immunoblot used for Fig. 2D
nSREBP2 (53 kDa) in nuclear fractions of HEK 293T cells activated by lipid depletion (NS – nonspecific bands).

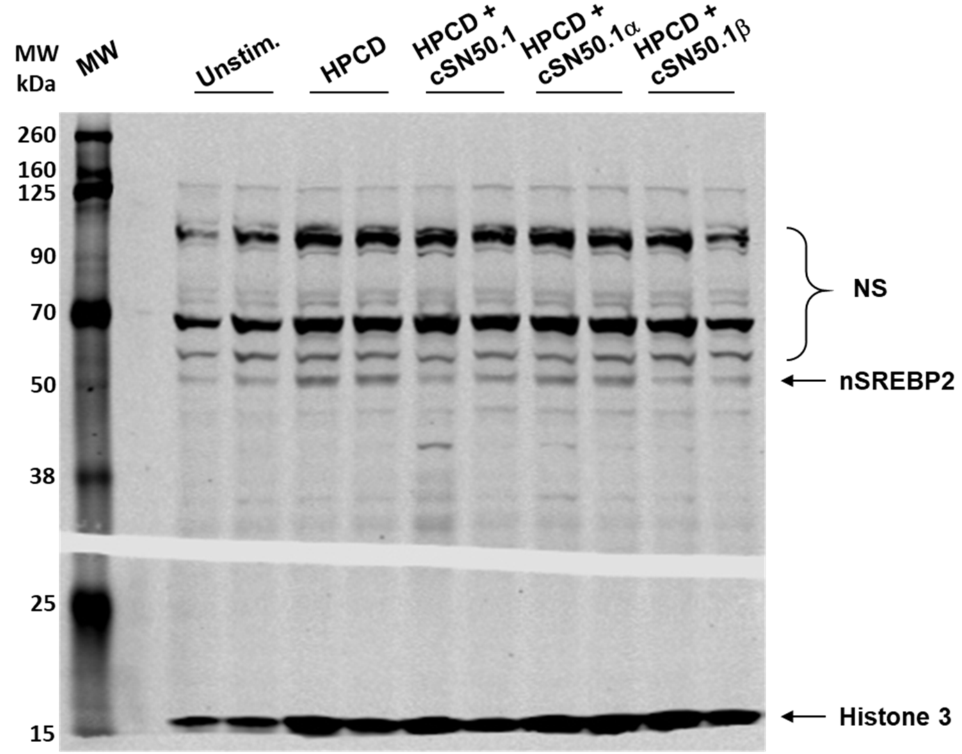

#

Suppl. Fig. S4E. Unedited immunoblot used for Fig. 2E
ChREBP (62 kDa splice variant) in nuclear fractions of HepG2 cells activated by Lo/Hi glucose + insulin.

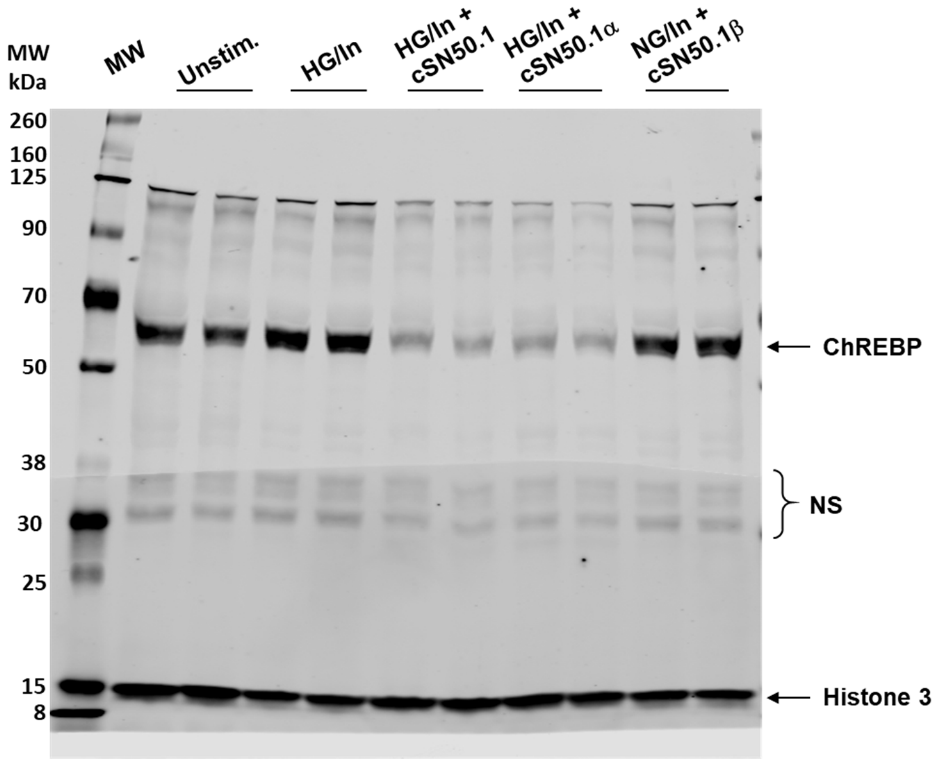

Supplementary Figure S5. Unedited full-length immunoblots used for preparation of Figures 4C and 6C.
All SDS PAGE electrophoreses were performed on 4% - 20% gradient gels and proteins content were transferred into membranes using Trans-Blot Turbo System (Bio-Rad). Membranes were then immunoblotted with the mix of primary antibodies: mouse monoclonal anti-TBP and rabbit monoclonal anti NF-κB RelA (p65) following by immunoblotting with the mix of secondary antibodies: goat anti-mouse IRDye 680RD (for detection of TBP in 700 nm channel) and donkey anti-rabbit IRDye 800CW (for detection of NF-κB RelA (p65) in 800 nm channel). After immunoblotting was completed, membranes were placed on the Odyssey CLx Infrared Imaging System (Li-Cor) and scanned with both channels. Images were quantitatively analyzed using Image Studio 3.1 software (Li-Cor).

#

Suppl. Fig. S5A. Unedited immunoblot used for Fig. 4C

NF-κB RelA (p65) in liver nuclear fractions of HFD-fed mice treated according to Long-Term Protocol.

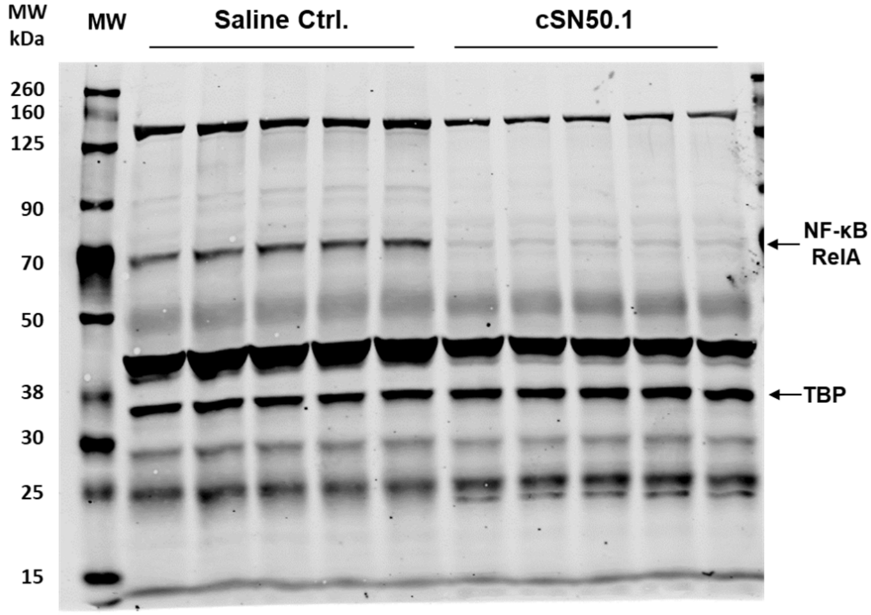

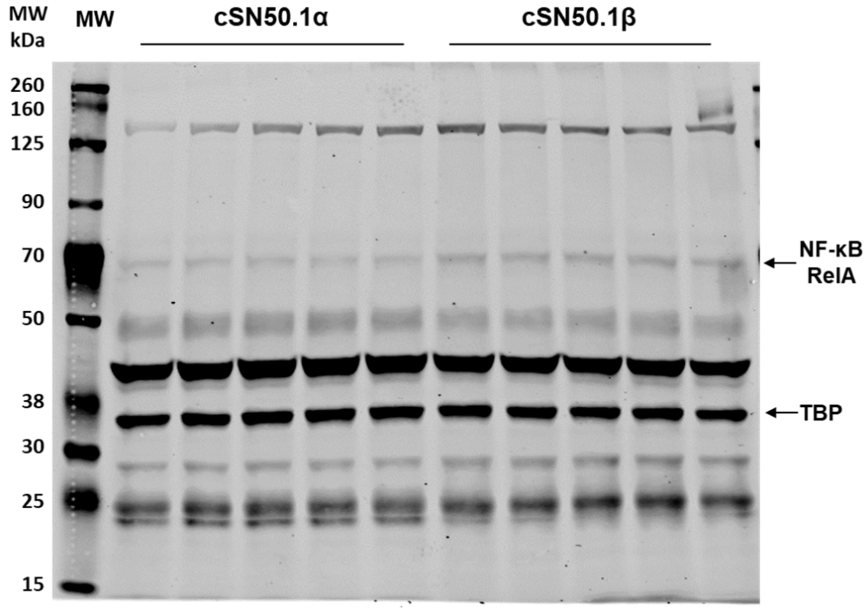

#

Suppl. Fig. S5B. Unedited immunoblot used for Fig. 6C

NF-κB RelA (p65) in liver nuclear fractions of HFD-fed mice treated according to Short-Term Protocol.

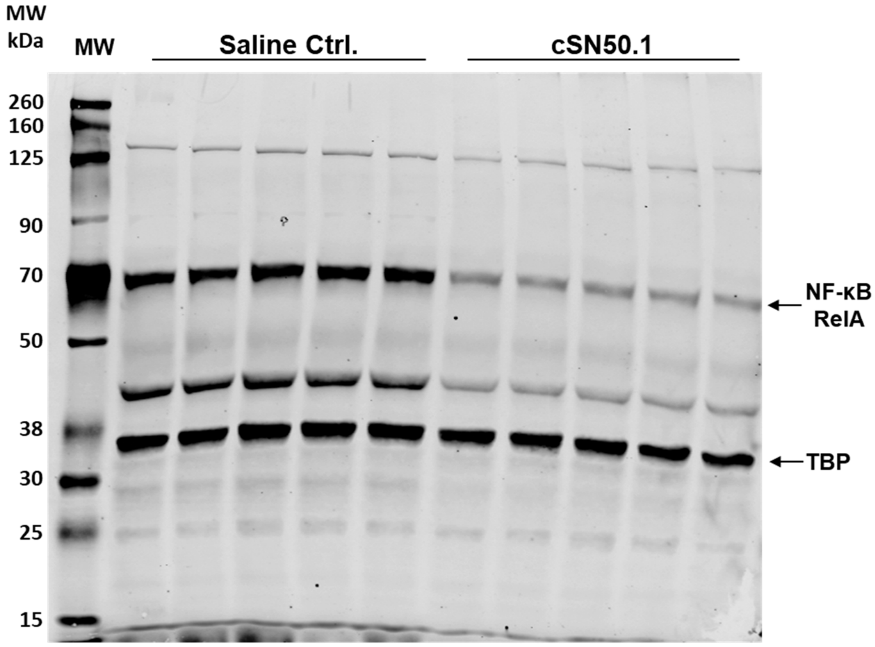

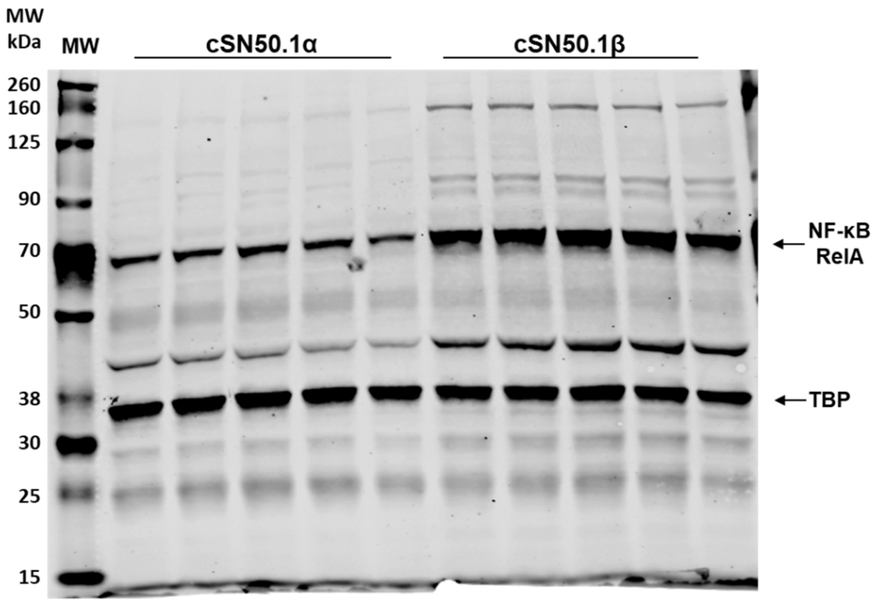

#
